# Genome-wide Analysis of bZIP Transcription Factors in wheat and Functional Characterization of a *TabZIP* under Abiotic Stress

**DOI:** 10.1038/s41598-019-40659-7

**Published:** 2019-03-14

**Authors:** Preeti Agarwal, Vinay Kumar Baranwal, Paramjit Khurana

**Affiliations:** 1 Department of Plant Molecular Biology, University of Delhi, South Campus, Benito Juarez Road, New Delhi, 110021 India; 2 Department of Botany, Swami Devanand Post Graduate College, Devashram Marg, Lar, Deoria, 274502 India

## Abstract

The basic leucine zipper **(**bZIP) represents one of the largest as well as most diverse transcription factor (TFs) families. They are known to play role in both stress as well as in various plant developmental processes. In the present study, a total of 191 bZIP transcription factors have been identified from *Triticum aestivum*. Expression analysis during various stress conditions, developmental stages, different varieties and gene ontology enrichment analysis suggest their possible roles in abiotic stress as well as in developmental responses. In the current analysis, one of the members named as *TabZIP* (Traes_7AL_25850F96F.1) was selected for detailed analysis to understand its role under different abiotic stress conditions. Gene expression studies revealed differential expression of *TabZIP* in various abiotic stress conditions like heat, salinity and dehydration suggesting the possible role of bZIP in various stress mitigation mechanism. *Arabidopsis* transgenics overexpressing *TabZIP* showed enhanced tolerance to salinity, drought, heat and oxidative stress. Thus *TabZIP* (Traes_7AL_25850F96F.1) can serve as a candidate gene for improving heat as well as other abiotic stress tolerance and can be helpful in enhancing the crop productivity under stress conditions.

## Introduction

Transcription factors are one of the most important regulators known to modulate gene expression in response to various environmental and developmental cues. Their role in mitigating various biotic and abiotic stress as well as developmental response is well studied. Among them bZIPs represent one of the largest and most diverse families and are uniquely present in eukaryotes. They range in numbers from 17 in *Saccharomyces cerevisiae*, 56 in *Homo sapiens*, 31 in *Caenorhabditis elegans*, 27 in *Drosophila melanogaster*, 89 in *Oryza sativa*, 92 in *Sorghum bicolor*, 89 in *Populus trichocarpa*, 67 in *Arabidopsis thaliana*, 96 in *Brachypodium distachyon*, 63 in *Sesamum indicum*, 121 in *Musa acuminata*, 77 in *Manihot esculenta* and 187 in *Triticum aestivum*^1–8^. They derive their name from a highly conserved bZIP domain consisting of a basic region and a less conserved leucine zipper domain^9^. The bZIP region consists of 40–80 amino acids where the basic region comprises of 16 amino acid residues and harbors a nuclear localization signal (NLS) followed by a unique invariant N-x7-R/K motif. The leucine zipper region consists of hepta repeats of leucine or other hydrophobic amino acids like isoleucine, valine, phenylalanine or methionine, located nine amino acids towards the C-terminal. The basic region is responsible for sequence specific DNA-binding activity while the leucine zipper is an amphipathic coiled-coil structure responsible for dimerization ability of bZIP. Only after the dimer is formed, it gains the ability to bind to the double stranded DNA at its major groove, through the basic region. Previous studies have suggested that plant bZIP proteins bind to DNA sequences with an ACGT core *cis*-element like ABRE, G-box (CACGTG), C-box (GACGTC), A-box (TACGTA), AACGTT (T box) and a GCN4 motif namely TGA(G/C)TCA, regulating the expression of downstream genes, thereby participating in diverse abiotic stresses including salinity, drought and cold^3,10–14^. The bZIP proteins are also well documented to play role in hormonal responses, light signaling and photomorphogenesis, seed germination, maturation, floral induction and flower development^15–19^. A large number of bZIP transcription factors have been functionally characterized, for instance role of *AtbZIP17* and *AtbZIP24* in salt stress in *Arabidopsis*^20,21^. In rice, functions of several members of bZIP family have been studied in great detail. For example *OsBZ8* and *OsbZIP16* play role in drought stress tolerance. *OsbZIP72* is known to play role in both ABA-dependent stress signaling and drought tolerance. *OsbZIP71* is reported to be strongly induced by drought, polyethylene glycol (PEG) and ABA treatments while its expression is repressed by salt treatment. Another *OsbZIP46* is found to be induced by drought, heat, hydrogen peroxide and ABA treatment. Similarly *OsbZIP60* is found to be positively regulated under heat and drought stress. *OsbZIP23* has been identified as transcriptional regulator in ABA-dependent signaling pathway which regulates expression of stress related genes in response to abiotic stresses. *OsbZIP52* was reported to be induced under low temperature of 4 °C. *OsABF1* was found to be induced by various abiotic stress treatments like anoxia, salinity, drought, oxidative stress, cold and ABA. It specifically expresses in seedling, shoots and roots^22–28^. In contrast to *Arabidopsis* and rice, only few bZIPs have been characterized in wheat. For example *WABI5* acts as a positive regulator of abiotic stress response^29^ while *TaABP1* from Chinese wheat landrace *Pingyaoxiaobaimai* (PYXBM) was observed to be strongly induced by ABA, high salt, low temperature and drought. Its expression was found to be higher in stems and leaves in comparison to the root tissue^30^.

Here in this manuscript, a concerted effort has been made to identify all bZIP coding genes from the most recent assembly of wheat genome (*Triticum aestivum*). One of the members of *TabZIPs* i.e. Traes_7AL_25850F96F.1 was used as candidate gene for functional characterization by transgenic approach and hereafter referred to as *TabZIP*. It was found to exhibit tissue and varietal specific expression. It also shows an induced expression in response to various abiotic stress treatments. Further, *Arabidopsis* transgenics overexpressing this particular bZIP show increased tolerance to salinity, dehydration and heat stress. Conclusively it can be inferred that *TabZIP* (Traes_7AL_25850F96F.1) can aid in overcoming heat stress induced ROS accumulation in transgenics as they show better performance in comparison to the wild type plants.

## Results

### Identification of bZIPs and analysis of their structural features

In our analysis, we have identified 195 proteins via hmm search. When these 195 proteins were examined for the presence of bZIP domain, four of them did not show the presence of this domain and was hence excluded from the analysis. Out of 191 identified bZIPs in the current version, 159 were present in the previously published report^8^. Thus in our analysis a total of 32 new members were identified. Out of 191 identified bZIPs, the average size of the transcript, CDS and protein length is ~3091 bp, ~760 bp and 252 amino acids respectively (Supplementary Fig. S1). The longest bZIP of transcript length of 12578 bp is encoded by Traes_5AS_3A85F1C2C.3. This gene codes for a CDS of 1173 bp with 12 exons. The smallest bZIP identified is Traes_2DS_D0563916C.1 with a cumulative length of 236 bp. The same gene has a CDS of 153 bp including 2 exons encoding for a 50 amino acid long protein. Traes_5DL_8F7AC72B0.2 has the maximum number of 18 exons with a modest size of 5261 bp of transcript. The average molecular mass for the family was calculated to be around ~20.62 KDa while the highest was 70.37 KDa for the gene with highest number of exons i.e. Traes_5DL_8F7AC72B0.2. A wider range of isoelectric focusing point for these bZIPs of wheat has been found. 142 bZIPs out of 191 identified were shown to have pI >7 and the rest 49 proteins have pI <7. Traes_2DS_D0563916C.1 has the maximum pI of 11.78 while Traes_7AL_25850F96F.1 has the lowest pI of 4.75. The detailed information of these bZIPs in terms of transcript, CDS, protein length, the number of exons, introns, molecular weight and other information has been provided in the Supplementary Table S1. It is possible that some of the genes identified may be pseudogenes and might have lost their functionality. However, structural analyses do not lead to identification of such genes in the identified set. Even in the global gene expression analyses using RNA-seq data establish that all identified genes are expressed. Thus, it may be a far reaching possibility that some of the identified genes could not lead to functional products and hence may act as pseudogenes.

Conserved motifs analysis using meme motif search led to identification of several motifs in these proteins (Supplementary Fig. S2 and Supplementary Table S2). All the proteins were predicted to have one or more bZIP domains. Besides this TGA like domains has been found in 29 genes, cAMP response element binding domain in 44, CCAAT-enhancer binding proteins (C/EBPs) domains in 22, coiled-coil domain in 40 and MFMR i.e. multifunction mosaic region in 11 bZIPs. In addition to these domains, three conserved domains of unknown function which could not be annotated were also identified. Domain of unknown function 1 (DUF1) with the conserved sequence has been identified in 20 bZIPs, DUF2 were present in eight such genes and DUF3 distribution was restricted to 27 out of 191 bZIPs. Multiple sequence alignment of identified proteins revealed the conserved ‘N-x7-R/K’ motif found in bZIP domain (Supplementary Fig. S3).

### Phylogenetic analysis of wheat bZIP proteins

In the current annotation of TAIR version 10.0, 75 *Arabidopsis* proteins having bZIP domains are present. Similarly in RGAP database there are 92 proteins having this bZIP domain. Rice proteins when searched for bZIP domain on NCBI-CDD database, 88 out of 92 proteins were found to posses this domain. Hence the bZIP domains from rice, *Arabidopsis* and wheat were fetched and were then used to align and generate an unrooted phylogenetic tree (Supplementary Fig. S4). Following the criteria proposed^31^ wheat bZIPs were classified in ten groups including Groups A, B, C, D, E, F, G, H, I and S. Ungrouped bZIPs in wheat were divided in three separated clades which were named as ungrouped 1, 2 and 3 respectively. All the members were separated well and formed distinct clades. *TabZIP* (Traes_7AL_25850F96F.1) falls in Ungrouped 3 (Supplementary Fig. S4). To understand the origin of these members in wheat itself, a separate bootstrapped NJ tree based on the whole protein alignment was prepared. In this analysis many of the gene pairs having strong bootstrap values and domains structural alignments were found. *TabZIP* (Traes_7AL_25850F96F.1) showed close similarity to Traes_7DL_3CE000E38.1 (Supplementary Fig. S2).

### Gene ontology enrichment analysis

Gene ontology (GO) enrichment analyses were performed for wheat bZIPs and it was found that a total of 48, 11 and 7 biological processes, cellular components and molecular function gene ontology terms were significantly (p value ≤ 0.05, hypergeometric distribution test) enriched (Supplementary Fig. S5, Supplementary Table 3–5). A few of these important GO terms from biological process include GO:0045449 (regulation of transcription), GO:0010468 (regulation of gene expression), GO:0019222 (regulation of metabolic process), GO:0010467 (gene expression), GO:0051254 (positive regulation of RNA metabolic process), GO:0009737 (response to abscisic acid stimulus), GO:0034641 (cellular nitrogen compound metabolic process) etc. Similarly, important gene ontology terms in cellular component category includes GO:0005634 (nucleus), GO:0043231 (intracellular membrane-bounded organelle), GO:0043227 (membrane-bounded organelle), GO:0043226 (organelle) and GO:0005622 (intracellular). Significantly enriched important molecular function GO terms for wheat bZIPs includes GO:0043565 (sequence-specific DNA binding), GO:0003700 (transcription factor activity), GO:0030528 (transcription regulator activity), GO:0003677 (DNA binding) and GO:0003676 (nucleic acid binding) apart from others.

### Identification of *cis*-acting elements in 1K upstream region

Transcription factors bind to the *cis*-elements present in the upstream regulatory region mainly to regulate the gene expression. Out of 469 *cis*-motifs defined in PLACE database, AAAG motif occurred the most which is recognized by DOF transcription factors. Other motifs like GTGA, CAAT, TGAC, GATA, AGAAA etc. were also frequently found in the putative promoter region of these bZIPs. Putative promoter region of only 153 genes could be fetched either due to non availability of upstream sequence in the contig or the starting of the gene from the extreme 5′ end of the contig. Based on the previous literature search depicting the putative function and expression domain, a set of 12 important *cis-*elements were identified in their 1K upstream region (Supplementary Fig. S6). Various *cis-*elements including those of Abscisic acid, Auxin, Ethylene and Gibberellic acid were identified. Abscisic acid response element (ABRE) occurred 37 times and their distribution was restricted to 31 genes. Moreover 63 ARF-binding sites were also identified in these promoters and were restricted to 51 of them. Seven Auxin Response Elements (AuxRE) were identified spanning seven bZIP promoters. 14 Gibberellic acid response elements (GARE) have been identified in the promoter of 12 genes. Central elements of Gibberellin response complex (GARC) were present in the promoter of 19 genes. Ethylene responsive elements were represented 36 times in these promoters and were distributed to 28 genes. DPBF1/2, a type of bZIPs binding core sequences was present 246 times distributed in 120 promoters. CCA1 binding sites (CBS elements) were present in the promoter of 14 genes suggesting their putative expression in circadian rhythm. CARG consensus box was present in the promoter of only one gene namely Traes_6DS_F8DFD036F.2. ARR-1 Binding site occurred 371 times in 136 bZIPs putative promoters. Low temperature response elements (LTRE) were found to be present 88 times in the promoter of 62 genes while 57 W Box were identified in 49 promoters.

### *TabZIPs* expression during development heat and abiotic stresses

Expression profile corresponding to 11 developmental stages like root, stem, leaves at 1 hr of HS, leaves at 6 hrs of HS, leaves exposed to combined stress of 1 hr of drought & heat, spike, stamen, pistil, grain and whole endosperm from various RNA-seq data has been obtained from wheat-expression.com^32^. Average of the normalized transcripts per million values was obtained from the database for each stage and stress. Out of 195 identified bZIPs, Traes_2BS_169BEF991.2 and Traes_7BL_096916DC5.1 have shown preferential accumulation in leaves challenged with 1 hr of heat stress. However, other bZIPs like Traes_7BL_625F55A12.1 and Traes_6AL_362B70E63.1 were found to express at a higher level in leaves subjected to combined stress of 1 hr of heat and drought treatment (Supplementary Fig. S7). Another *TabZIP*, Traes_2AS_B1B372658.2 showed similar expression level in response to HS for 1 hr, HS for 6 hr and combined stress of drought and heat for 1 hr while TRAES3BF019000220CFD_t1 and Traes_6AL_362B70E63.1 showed similar expression in response to HS for 1 hr and combined stress of drought and heat for 1 hr.

Besides their role in abiotic stress, *TabZIPs* also showed tissue specific expression as observed from Supplementary Fig. S7. Traes_1AS_94B6230FB1.1, Traes_5DS_011851E E7.1, Traes_1DS_62FDD3493.1, Traes_1BS_A44C97E0F.2 and Traes_5BS_ FF44610EF 1.3 expressed in all tissue with maximum expression in root. Similarly, Traes_5DS_011851EE7.1, Traes_1AS_94B6230FB1.1, Traes_5BS_FF44610EF 1.3, Traes_1DS _62FDD3493.1 and Traes_6DS_273430303.2 showed preferential expression in stem tissue. In addition to this, the expression of Traes_5DS_011851EE7.1, Traes_1AS_94B6230FB1.1, Traes_5BS_FF44610EF1.3, Traes_1DS _62FDD3493.1 were found to be quite significant in other tissues like spike, stamen and pistil (Supplementary Fig. S7). Moreover, 5DS_011851EE7.1 and Traes_5BS_FF44610EF1.3 showed a high transcript level in grain and whole endosperm tissue and was also found to express under heat stress as evident from high expression level in leaves after both 1 hr and 6 hr heat stress. Other bZIPs like Traes3BF111600130CFD_t1, Traes_4DL_268C3D168.1, Traes_4AL_792F1D482.1, Traes_5DL_75AEE92BB.1 and Traes_5BL_F3018E8CA.1 displayed low level of expression with slight increase in expression level in grain and whole endosperm. Moreover, the expression of one of the *TabZIP* i.e. Traes_7AL_25850F96F.1 selected for a detailed analysis was found to be higher in roots, stamen and pistil. Besides this, its expression was also induced in leaves conditioned to heat stress for 1 hr and 6 hr. In addition to heat stress, it was also found to be induced under combined drought and heat stress for 1 hr (Supplementary Fig. S7).

### Expression analysis of bZIPs in wheat varieties

Using the RNA-seq data in public resources we have compared the expression profile of bZIPs in 13 wheat varieties namely Bobwhite, Sevin, N9134, TAM107, Banks, P271, Synthetic Hexaploid, Chinese Spring, Holdfast, HTS-1, CM-82036 and Avocet S (Supplementary Fig. S8). Out of all the bZIPs, Traes_7DL_68B814464.1, Traes_6BS993CBD840.1, Traes_6AS_AFF47E71C.2, Traes_7AL_D89BDD619.1 and Traes_7AS_FDD164762.2 showed highest expression in Sevin. Traes_5DS_011851EE7.1 was found to express at higher level in all the above varieties while the transcript of Traes_5BS_FF44610EF1.3 and Traes_1DS_62FDD3493.1 was found to be high in all cultivars except in Sevin. bZIPS like Traes_4BL_4C9A415F3.1, Traes_2BL_709E67A02.1, Traes_2DL_5610BA574.1 and Traes_7BS_F7433ACA4.1 showed maximum expression in N9134. Traes_2DS_D0563916C.1 and Traes_2AS_ 6EA1E372F.1 shows highest expression in synthetic hexaploid variety (Supplementary Fig. S8). Another bZIP Traes_3AL_FC5523394.2 was found to express specially in BANKS and Holdfast with the highest expression level observed in BANKS. Traes_7DS_47B5A7FFF.1, Traes_6BS_993CBD840.1, Traes_6AS_AFF47E71C.2, Traes_7DL_70D4FDB2A.1, Traes_7BL_096916DC5.1 and TRAES3BF099600120CFD_t1 have high expression in Bobwhite variety with the highest transcript level observed for Traes_7DL_70D4FDB2A.1. Moreover, the expression of one of the bZIPs i.e. Traes_7AL_25850F96F.1 selected for detailed analysis was found to be high in HTS1, Avocet S, BANKS, TAM107, P271, Cold Spring and Hold fast with the maximum expression in HTS1, Avocet S and BANKS (Supplementary Fig. S8). Thus, each bZIP displays a unique expression profile thereby suggesting a varietal specific response of these bZIPs.

### *TabZIP (Traes_7AL_25850F96F*.*1)* expression profile under various stress and developmental stages

**Figure 1:**
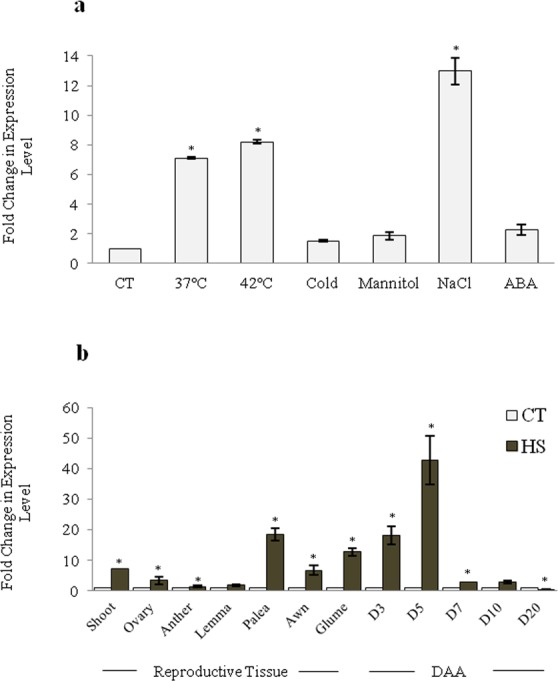
Expression profiling of *TabZIP* transcription factor using real-time PCR in response to various stresses. Change in the transcript level of *TabZIP* in (**a)** Ten days grown wheat seedlings of PBW343 in response to heat, cold, salt, mannitol and ABA treatment. (**b)** In shoot and reproductive tissues (from mature plant) of PBW343 after heat stress. The expression level of control tissue (0 h of treatment) was normalized as 1.0. Results obtained are the mean ± standard deviations of minimum three independent experiments. CT-Control, HS-Heat Stress, DAA (Days After Anthesis)-D3, D5, D7, D10, D20). *Asterick represents the significant difference i.e. Student’s t-test, P- value of ≤0.05.

To understand the role of *TabZIPs* under abiotic stress conditions, expression profiling of Traes_7AL_25850F96F.1 (hereafter referred to as *TabZIP*), was undertaken in ten days old seedlings of PBW343 and subjected to heat stress, at both 37 °C and 42 °C for two hrs (Fig. 1a). It was selected since this particular bZIP was also identified in heat stress subtractive hybridization library where it showed a preferential expression in floral organs especially in anther and ovary^33^. Therefore, it was used as a candidate gene for understanding the molecular mechanism under heat stress as well as in other abiotic responses. From real-time PCR analysis it was found to be up-regulated (>4-folds) under both basal and high temperature stress. *TabZIP* was also observed to be up-regulated under various stress treatments with maximum level of expression under salt stress (>10-folds) followed by ABA treatment (Fig. 1a). Therefore such a differential expression under dehydration, salt, cold and ABA treatments suggest possible role of *TabZIP* in various abiotic stress response. Further analysis was undertaken to examine tissue specific response of *TabZIP* under heat stress. Therefore, expression profiling was examined in various reproductive tissues including anther, ovary, glumes, palea, lemma, awn and at various stages of seed development at different days after anthesis (DAA) like 3, 5, 7, 10 and 20DAA. This study revealed that *TabZIP* expresses highest in seed at 5DAA (>40-fold changes) followed by 3DAA (>20 fold change) and in palea tissue with >20 fold change in expression level (Fig. 1b).

### Heat stress mediated expression dynamics of *TabZIP* (Traes_7AL_25850F96F.1) in sensitive and tolerant varieties

**Figure 2:**
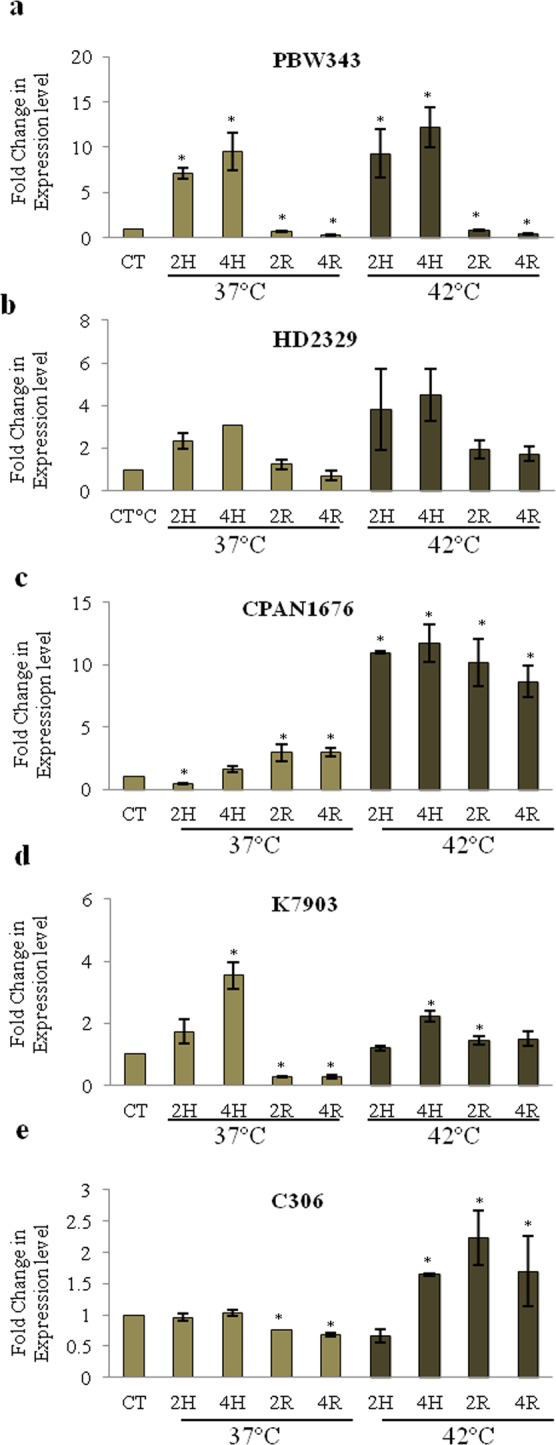
Expression analysis of *TabZIP* transcription factor in response to heat stress in five varieties of *Triticum aestivum*. (**a)** PBW343, (**b)** HD2329, (**c)** CPAN1676, (**d)** K7903 and (**e)** C306. Seedlings were given heat stress (37 °C and 42 °C) for two hrs, followed by four hrs of recovery. The expression level of *TabZIP* of control sample (0 h of treatment) was normalized to 1.0. Results obtained are mean ± standard deviations of minimum three independent experiments. *Asterick represents the significant difference i.e. Student’s t-test, P- value of ≤ 0.05.

In order to understand the varietal specific respone under heat stress, the expression of *TabZIP* was analysed in heat sensitive and tolerant varieties. For this PBW343 and HD2329 (heat sensitive varieties), a moderately tolerant CPAN1676 variety and C306 and K7903 (heat tolerant varieties) were used according to study by Hairat *et al*.^34^. Ten day old seedlings of these varieties were given heat stress (37 °C and 42 °C) for two hrs and four hrs and then followed by recovery (two hrs and four hrs). In PBW343 and HD2329, the expression of *TabZIP* was found to be up-regulated at 37 °C and 42 °C and shows a slightly increased expression level during recovery almost near to control tissue (Fig. 2a,b). In CPAN1676, the expression level of *TabZIP* after 37 °C remains unchanged with slight increase during the recovery period. However, at 42 °C the expression was greater than four-fold change (Fig. 2c). The expression of this bZIP in tolerant varieties was not found to be regulated differentially in response to any heat stress treatments except in K7903 (Fig. 2d,e). Thus these observations suggest that *TabZIP* is regulated in a varietal specific manner under different stress treatment regime.

### Transactivational activity assay of *TabZIP*

**Figure 3:**
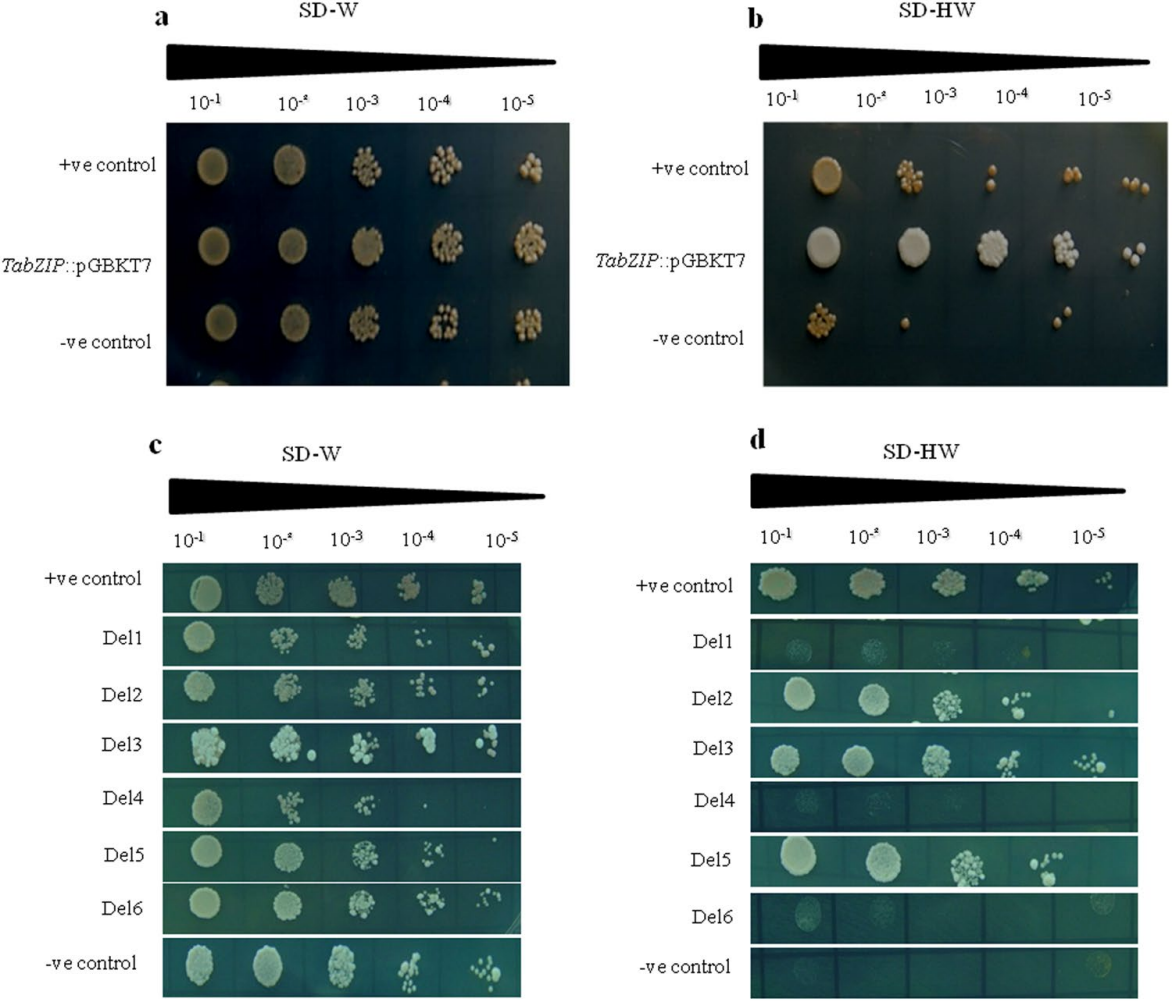
Transcription activation assay of TabZIP in yeast. Growth of yeast AH109 strain harbouring full length CDS of *TabZIP*::pGBKT7 constructs along with both positive and negative controls on (**a)** SD-H and (**b)** SD-HW media. Growth of yeast AH109 strains containing various deletions construct of *TabZIP*::pGBKT7 on (**c)** SD-W and (**d)** SD-HW media.

To test the transactivation potential of TabZIP, yeast hybrid assay system was employed. The yeast harboring pGBKT7::*TabZIP* grew on histidine lacking medium while the empty vector control showed no growth (Fig. 3a,b). Various deletion constructs were prepared in order to delineate the function of its various domains in conferring trans-activation potential. It was observed that Del-1 (only N-terminal), Del-4 (N-terminal along with bZIP domain) and Del-6 (both N-terminal and C-terminal without bZIP domain) did not show transactivation activity while Del-2 (only c-terminal), Del-3 (C-terminal with bZIP domain) and Del-5 (only bZIP domain) showed the transactivation activity (Fig. 3c,d). Thus TabZIP has a transactivation potential and its transactivity resides in the C-terminal region. Moreover, BRLZ domain too posses the transactivation activity since BRLZ domain alone is also found to show transactivity as seen by growth of construct Del-5. In addition to this, the presence of N-terminal was found to inhibit the transactivity of TabZIP. This result is in corroboration with AtbZIP68 where N-terminal region serves as a transcriptional repressor^35^ and OsbZIP23 where shortened region from both the N and C terminal posses transactivity^22^.

### *TabZIP* confers tolerance to salinity stress

**Figure 4:**
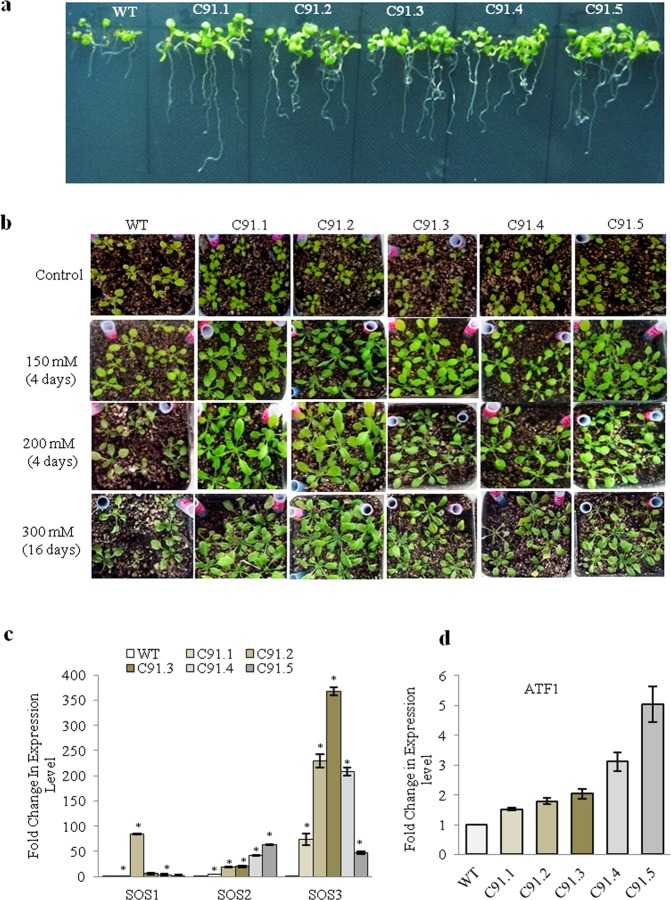
*TabZIP* over expressing *Arabidopsis* plants showing increased tolerance to salt stress. (**a)** Effect of salt stress on *TabZIP* overexpression *Arabidopsis* transgenic lines subjected to stress by supplementing media with 150 mM NaCl and phenotype was observed after twenty days. (**b)** Effect of salt stress on 14-day-old *TabZIP* over-expressing transgenic lines of *Arabidopsis* subjected to salt solution treatments i.e. 150 mM NaCl for four days followed by 200 mM NaCl for another four days and then subjected to 300 mM NaCl for sixteen days. (**c)** Relative abundance of *SOS1*, *SOS2*, *SOS3* and (**d)** *ATF1*. The expression level of wild type (0 h of treatment) was normalized to 1.0. Results depicted are mean ± standard deviations of minimum three independent experiments. *Asterick represents the significant difference i.e. Student’s t-test, P- value of ≤ 0.05.

To find out the role of *TabZIP*, *Arabidopsis* transgenic lines overexpressing *TabZIP* (Traes_7AL_25850F96F.1) were raised. The transgenics showed high level of *TabZIP* expression in comparison to wild type (Supplementary Fig. S9). To analyze the response under salt stress, both wild type and transgenic seedlings were grown on half MS media supplemented with 150 mM NaCl. Under salt stress, overexpression lines were found to be considerably healthy and grew well in comparison to wild type (Fig. 4a). These transgenics also showed relatively higher fresh weight and longer root with respect to the wild type (Table 1). Proline level was also analysed since its accumulation is known to be an early indicator as an osmoprotectant in response to salt stress^36^. Proline content was found to be substantially more in transgenics (Table 1). Other parameters including Fv/Fm ratio indicating the photosynthetic efficiency and membrane stability index were also found to be higher (Table 1).

**Table 1:** *TabZIP* overexpressing *Arabidopsis* plants showing increased tolerance to salinity.

Parameters	WT	C91.1	C91.2	C91.3	C91.4	C91.5
**Seedling stage (150** **mM of NaCl)**						
Root Length (cm)	0.35 ± 0.07	1.11 ± 0.23*	1.30 ± 0.10*	1.35 ± 0.12*	1.41 ± 0.09*	1.41 ± 0.09*
Fresh Weight (mg)	2.22 ± 0.06	6.40 ± 0.01*	4.12 ± 0.01*	5.71 ± 0.02*	6.87 ± 0.01*	7.23 ± 0.02*
Photosynthetic Efficiency (Fv/Fm)	0.52 ± 0.06	0.64 ± 0.01	0.68 ± 001	0.70 ± 0.02	0.73 ± 0.01	0.66 ± 0.01
Proline Content (mg/ml)	0.99 ± 0.17	2.81 ± 0.22	3.38 ± 0.28	6.27 ± 0.58	4.94 ± 0.23	5.49 ± 0.23
Membrane Stability (%)	0.26 ± 0.03	0.35 ± 0.03	0.33 ± 0.01	0.35 ± 0.04	0.33 ± 0.03	0.27 ± 0.03
**Mature stage (Gradual salt stress)**						
Leaf Diameter (cm)	2.53 ± 0.18	5.33 ± 0.04*	4.13 ± 0.08*	5.35 ± 0.15*	3.95 ± 0.23*	3.9 ± 0.17*
Leaf Length (cm)	0.71 ± 0.11	1.63 ± 0.06*	1.15 ± 0.06*	1.71 ± 0.07*	1.28 ± 0.04*	1.28 ± 0.06*
Leaf Breadth (cm)	0.58 ± 0.05	0.9 ± 0.04*	0.85 ± 0.02*	0.95 ± 0.03*	0.88 ± 0.03*	0.93 ± 0.04*
Fresh Weight (mg)	0.02 ± 02	0.07 ± 0.003*	0.07 ± 0.01*	0.08 ± 0.01*	0.06 ± 0.01*	0.05 ± 0.16*
Proline Content (mg/ml)	1.89 ± 0.15	2.81 ± 0.32	3.38 ± 0.31*	6.27 ± 0.94*	4.94 ± 0.67*	5.49 ± 0.11
Chlorophyll Content (mg/ml)	2.72 ± 0.01	4.15 ± 0.05*	5.16 ± 0.02*	3.34 ± 0.04*	4.7 ± 0.003*	3.88 ± 0.31

*Represent P-value of ≤0.05; ±sign represent standard error.

Since the transgenics were found to perform well under 150 mM salt stress, further research was conducted to examine growth of wild type and transgenics through a gradual increase in salt concentration. For this, two-week-old seedlings were initially watered with 150 mM NaCl solution for four days followed by 200 mM NaCl for another four days, and were finally subjected to 300 mM NaCl for sixteen days.

The seedlings (transgenics and wild type) grew well under 150 mM NaCl. However with the increase in the concentration of NaCl, the wild type turned pale and showed poor growth while the transgenics showed faster and healthier growth under stress conditions (Fig. 4b). After twenty four days of stress the transgenics showed enhanced fresh weight, larger leaf lamina, higher chlorophyll and proline content (Table 1). Moreover, the expression of salt stress marker genes including *SOS1*, *SOS2*, *SOS3* and *ATF1* was also notably higher in transgenics than wild type plants (Fig. 4c,d).

### *TabZIP* increases tolerance and yield under drought stress

**Figure 5:**
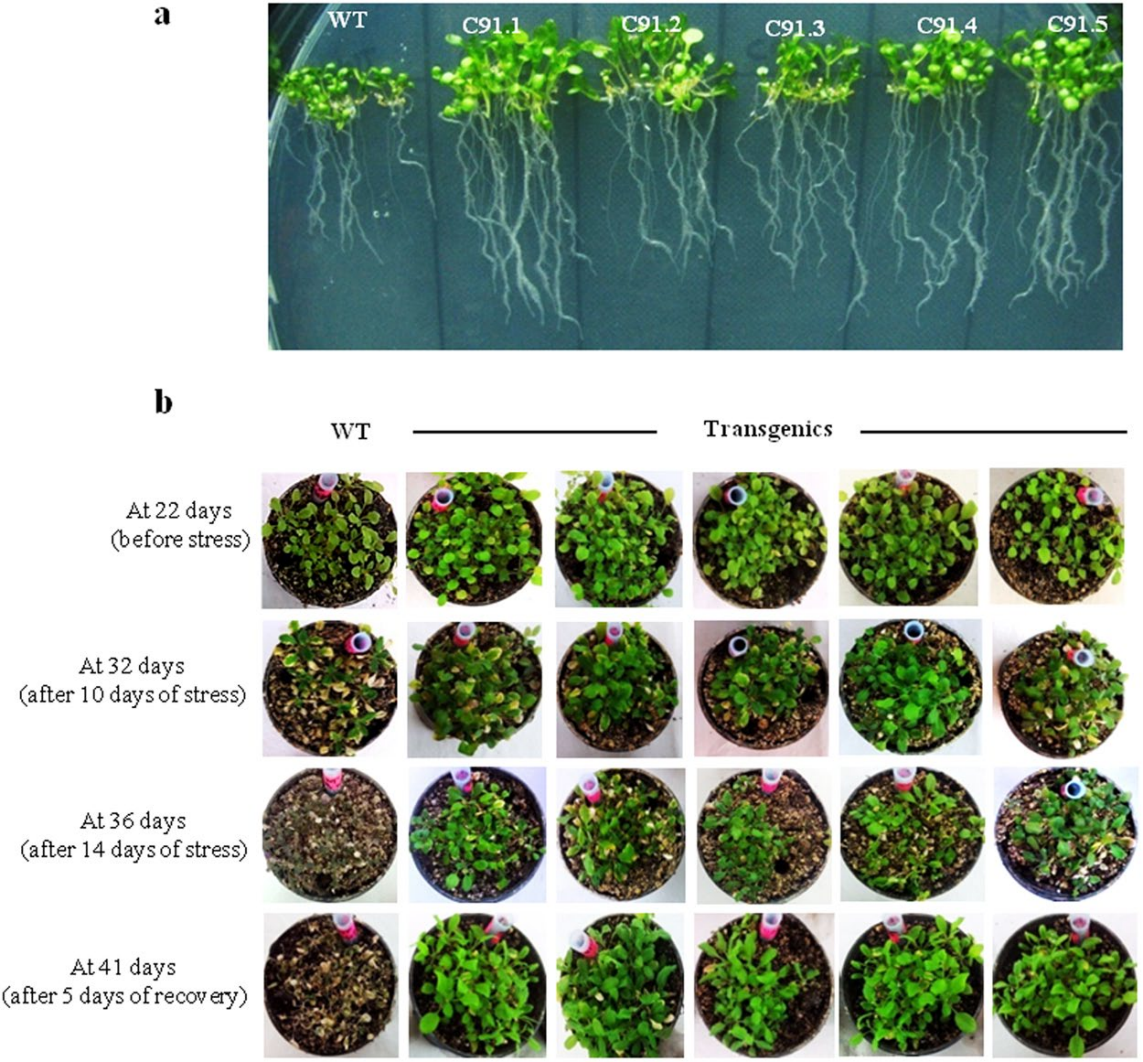
*TabZIP* overexpression plants showed enhanced tolerance to dehydration stress. (**a**) Effect of dehydration on *TabZIP* overexpression *Arabidopsis* transgenic lines subjected to stress by supplementing media with 4% PEG and phenotype was observed after twenty days. (**b)** Effect of drought stress by subjecting 22-days-old wild type and transgenic seedlings to drought treatment for fourteen days and then followed by recovery i.e. rewatering the plants for five days.

**Figure 6:**
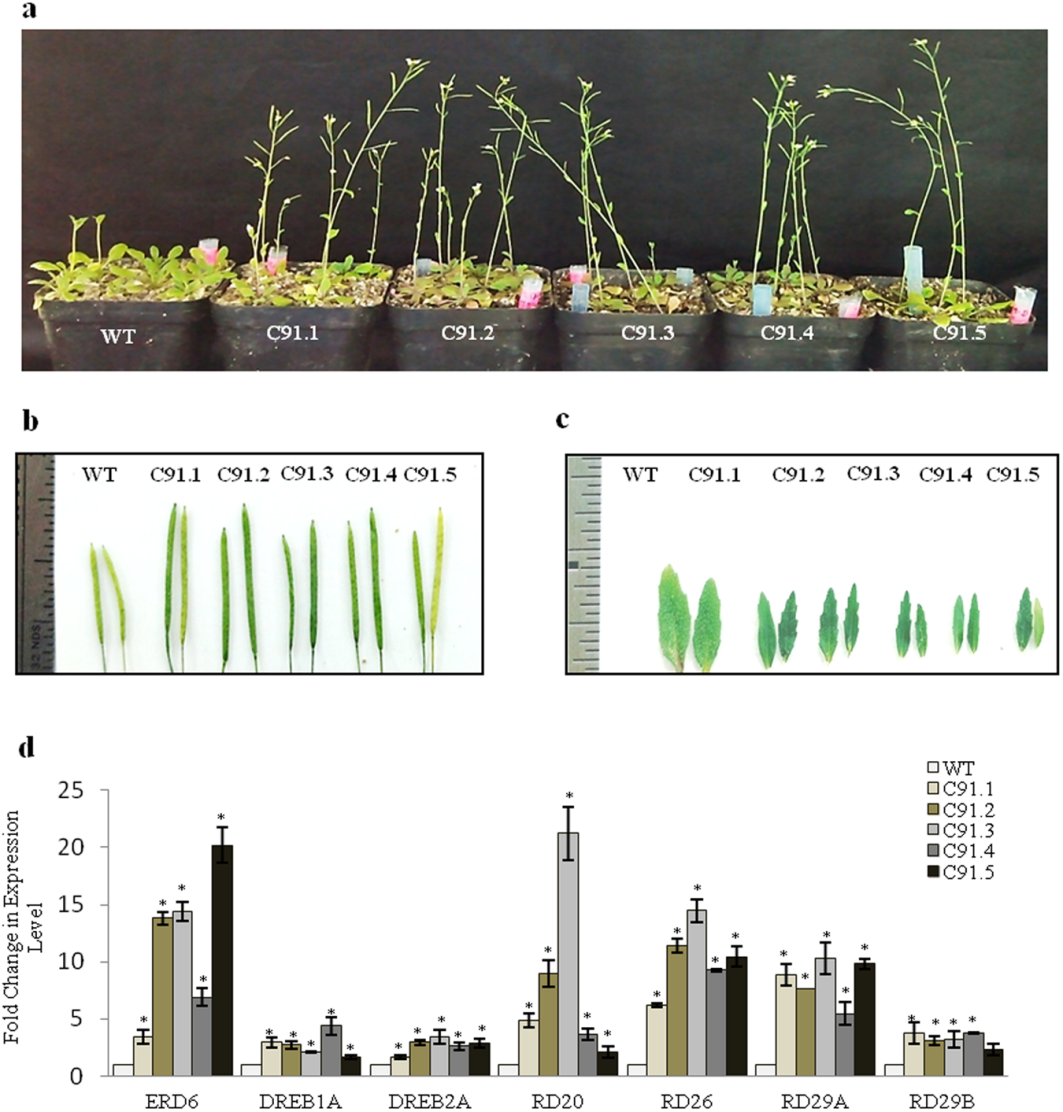
*TabZIP* overexpression plants showed enhanced tolerance to drought stress. Effect of drought stress on transgenic lines (one month old) subjected to drought stress for almost one month. (**a)** Phenotype of wild type and transgenic plants observed after stress, (**b)** size of siliques, (**c)** leaf morphology and (**d)** relative transcript abundance of drought stress marker gene in wild type and *TabZIP* overexpression transgenics. Expression level in wild type was normalized to 1.0. Results obtained are mean value ± standard deviations of minimum three independent experiments. *Asterick represents the significant difference i.e. Student’s t-test, P- value of ≤0.05.

To find out the possible role of *TabZIP* in response to drought stress, both wild type and transgenics were grown on MS media supplemented with 4% PEG. The transgenics showed longer roots, robust growth and more fresh weight (Fig. 5a; Table 2). Previous research reports suggest that salinity and drought stress cause substantial damage to photosynthetic pigments^37^. The transgenics showed better response with regard to their Fv/Fm, proline content and membrane stability (Table 2). Since transgenics were found to perform better under dehydration stress we further analyzed the overall growth of transgenics under drought stress. Both wild type and transgenic lines were grown in soilrite for 22 days in small plastic pots in culture room followed by the onset of drought by removal of water for next 14 days. Observations were noted till 14 days of stress treatment. Transgenics were found to be more tolerant as they survived as well as showed robust growth, whereas the wild type seedlings perished under drought stress conditions. Even after rewatering, the wild type did not recover while the transgenics recovered well and showed healthier and profuse growth (Fig. 5b; Table 2). Since transgenics were found to perform better under drought stress, further experiments were conducted to examine the effect of drought on overall growth and yield of the plant. For this, seedlings were grown for a month under normal conditions and then subjected to drought treatment by not watering the plants for almost a month. The transgenics were found to grow well and showed early flowering eventually leading to early seed formation and higher yield in comparison to the wild type. Siliques of overexpression transgenics were larger in size and more in number (Fig. 6a,b; Table 3). The leaves of these plants were smaller than wild type. This decrease in leaf size is one of the most common mechanisms to escape or overcome stress conditions (Fig. 6c; Table 3). Moreover, this phenotype also indicates the mobilization of food resulting in early seed setting which is one of most important feature of plants exposed to stress conditions. This further substantiates with the early flowering and seed formation phenotype as observed in transgenics (Fig. 6a,b). The transgenics also showed higher proline and chlorophyll content (Table 3). The expression of various drought stress marker genes like *ERD6*, *DREB1A*, *DREB2A*, *RD20*, *RD26*, *RD29A* and *RD29B* were analyzed so as to understand their regulation under stress conditions. The transcript level of all these marker genes were considerably higher in transgenics in comparison to wild type (Fig. 6d).

**Table 2:** *TabZIP* overexpressing *Arabidopsis* plants under dehydration stress.

Seedling Parameters	WT	C91.1	C91.2	C91.3	C91.4	C91.5
Root Length (cm)	1.44 ± 0.11*	2.48 ± 0.04*	2.34 ± 0.12*	2.38 ± 0.13*	2.68 ± 0.15*	2.52 ± 0.09*
Fresh Weight (mg)	5.30 ± 0.38*	7.53 ± 0.06*	7.30 ± 1.33*	8.60 ± 0.69*	8.93 ± 0.60*	5.82 ± 0.29
Photosynthetic Efficiency (Fv/Fm)	0.60 ± 0.04*	0.76 ± 0.11*	0.76 ± 0.15*	0.77 ± 0.022*	0.77 ± 0.005*	0.73 ± 0.005*
Proline Content (mg/ml)	2.57 ± 0.07	3.87 ± 0.28	3.72 ± 0.35	4.18 ± 0.12	3.09 ± 0.30	3.39 ± 0.34
Membrane Stability Index (%)	0.39 ± 0.02	0.52 ± 0.4*	0.54 ± 0.03*	0.48 ± 0.02*	0.50 ± 0.02*	0.55 ± 0.01*
Survival Rate (%)	0.00 ± 0.00	16.75 ± 0.63*	17.25 ± 0.47*	16.50 ± 0.29*	17.00 ± 0.40*	16.75 ± 0.63*

*Represent P-value of ≤0.05; ±sign represent standard error.

**Table 3:** *TabZIP* overexpressing *Arabidopsis* plants showing increase tolerance to drought stress.

Mature Plants	WT	C91.1	C91.2	C91.3	C91.4	C91.5
Silique Length (cm)	1.10 ± 0.05	1.60 ± 0.08*	1.50 ± 0.06*	1.70 ± 0.05*	1.70 ± 0.05*	1.70 ± 0.08*
No of siliques per plant	3.00 ± 0.5	10.00 ± 0.5*	12.00 ± 1.2*	14.00 ± 1.2*	11.00 ± 1.2*	12.00 ± 0.8*
Seed yield per plant (mg)	0.91 ± 0.91	3.14 ± 0.33*	3.43 ± 0.32*	5.34 ± 0.48*	3.94 ± 0.24*	3.61 ± 0.64*
Plant Height (cm)	5.67 ± 0.68	12.23 ± 0.50	12.83 ± 0.50*	13.67 ± 0.50*	10.8 ± 0.50	13.77 ± 1.49*
Leaf Length (cm)	0.50 ± 0.05	0.30 ± 0.03	0.40 ± 0.05	0.40 ± 0.08	0.30 ± 0.08	0.40 ± 0.03
Leaf Breadth (cm)	0.30 ± 0.03	0.10 ± 0.01	0.20 ± 0.03	0.20 ± 0.05	0.20 ± 0.05	0.20 ± 0.05
Proline Content (mg/ml)	0.27 ± 0.02	0.83 ± 0.04*	0.82 ± 0.03*	0.79 ± 0.10*	0.79 ± 0.06*	0.50 ± 0.02*
Chlorophyll Content (mg/ml)	1.75 ± 6.07	0.07 ± 0.07*	7.86 ± 0.16*	5.82 ± 0.01*	6.05 ± 0.04*	3.30 ± 0.05*

*Represent P-value of ≤0.05; ±sign represent standard error.

### *TabZIP* enhances thermotolerance

**Figure 7:**
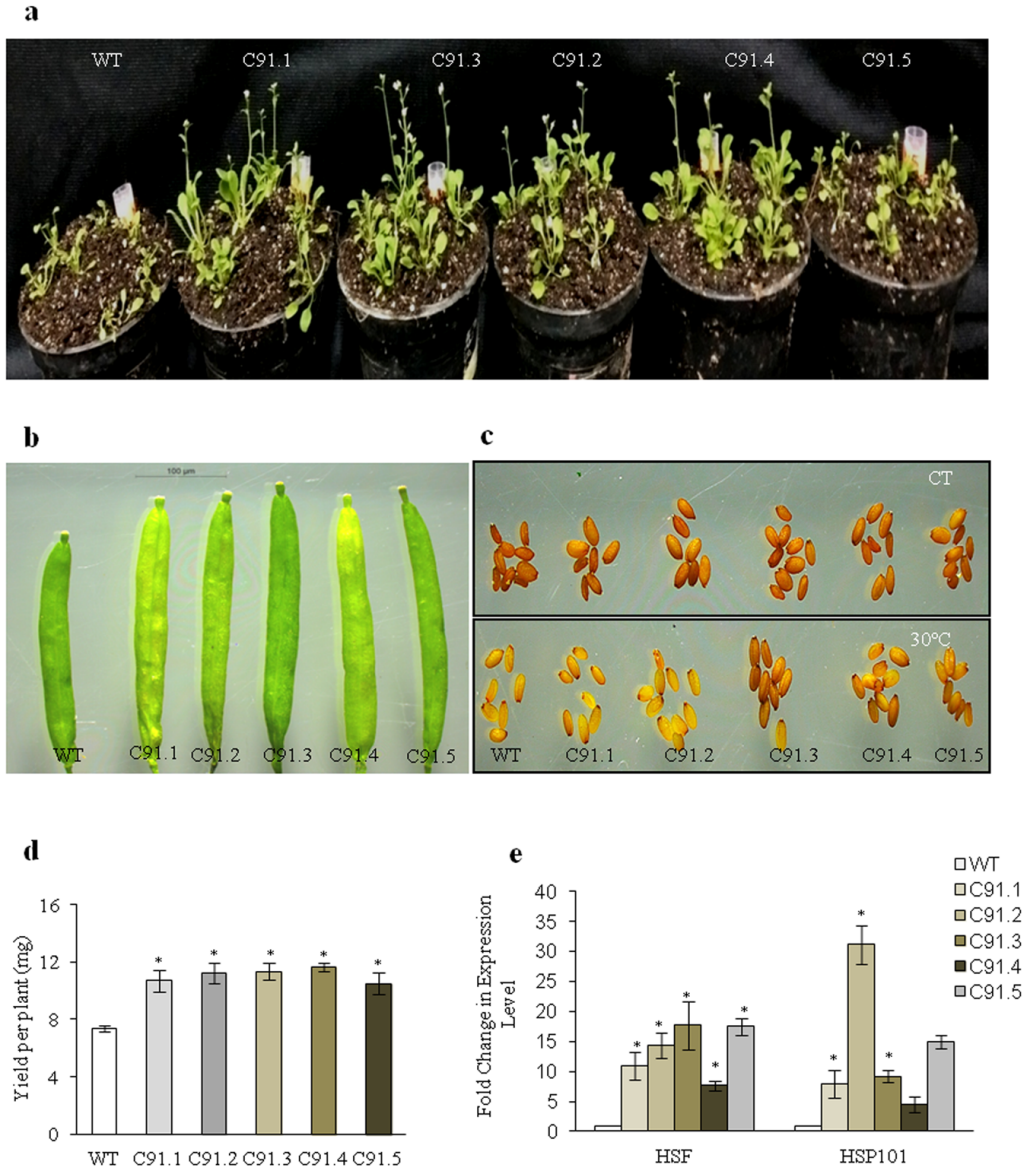
*TabZIP* overexpressing *Arabidopsis* plants enhances thermotolerance. *Arabidopsis* transgenic harboring *TabZIP* under constitutive promoter showed better response to heat stress treatment (42 °C) for two hrs in comparison to the wild type. **(a)** Phenotype of plants post recovery at 20 days. (**b)** Silique size. (**c)** Seed morphology assay of WT and *TabZIP* overexpressing transgenics of *Arabidopsis*. (**d)** Yield per plant. (**e)** Relative transcript abundance estimation of heat stress marker gene (HSF) in wild type and *TabZIP* overexpression transgenic plants. The expression level of wild type was normalized to 1.0. Results obtained are mean value ± standard deviations of minimum three independent experiments. *Asterick indicates the significant difference i.e. Student’s t-test, P- value of ≤0.05.

Since *TabZIP* is up-regulated in response to heat treatment in wheat varieties PBW343 and HD2329, overexpression lines were also examined for their role in heat stress at 42 °C (for two hrs) followed by recovery. The transgenics revived well as observed after 20 days of recovery period (Fig. 7a) and showed faster and robust growth thereafter. They displayed healthier growth as measured by their root length, rosette diameter, leaf number and its size (Table 4). They showed higher yield as indicated by their number of siliques per plant (Table 4), size of siliques and the total yield (per plant) (Fig. 7b,d).

**Table 4:** Morphological analysis of *TabZIP* overexpressing *Arabidopsis* plants showing increase tolerance to heat stress.

Parameters	Plant Height (cm)	Shoot Height (cm)	Root Length (cm)	Rosette Diameter (cm)	Leaf Numbers	Leaf Length (cm)	Leaf Breadth (cm)	Fresh Weight (mg)	No. of siliques per plant
WT	1.30 ± 0.01	0.20 ± 0.01	1.21 ± 0.08	0.49 ± 0.07	8.1 ± 0.18	0.12 ± 0.02	0.08 ± 0.11	0.01 ± 0.003	10 ± 1.15
C 91.1	3.70 ± 0.05*	2.07 ± 0.03*	2.33 ± 0.15*	2.10 ± 0.08*	22.77 ± 1.48*	0.65 ± 0.04*	0.54 ± 0.07*	0.22 ± 0.02*	62.33 ± 1.45*
C 91.2	3.30 ± 0.03 *	1.47 ± 0.08*	1.71 ± 0.19	2.40 ± 0.40	22.53 ± 5.52	0.45 ± 0.02*	0.42 ± 0.01*	0.32 ± 0.03*	65.33 ± 2.90*
C 91.3	3.20 ± 0.02 *	2.39 ± 0.10	1.49 ± 0.20	2.42 ± 0.13*	23.85 ± 2.21*	0.43 ± 0.10*	0.32 ± 0.06	0.22 ± 0.04	57.66 ± 1.45*
C91.4	3.35 ± 0.08*	2.28 ± 0.05*	1.51 ± 0.31	2.21 ± 0.50	22.32 ± 2.11	0.42 ± 0.41	0.35 ± 0.08	0.23 ± 0.04*	58.0 ± 2.08*
C91.5	4.12 ± 0.03 *	2.34 ± 0.04*	1.36 ± 0.02	2.22 ± 0.10	20.21 ± 12	0.52 ± 0.23	0.36 ± 0.02*	0.26 ± 0.01*	57 ± 1.15*

*Represent P-value of ≤0.05; ±sign represent standard error.

Photosynthesis is known to be highly sensitive to high temperature stress^38^. Therefore, heat stress effect was analyzed by studying several photosynthetic parameters like Fv/Fm, ETR and YII. The transgenics were found to perform better than wild type plants with respect to these parameters (Table 5). In addition to this, membrane stability was also analyzed since membrane is known to be primary site of physiological injury^39^. The transgenics under heat stress showed better membrane stability and higher proline content than wild type seedlings (Table 5). Expression level of heat stress marker genes e.g. *HSF* and *HSP101* was also examined under heat stress conditions where it was comparatively higher in transgenics than wild type (Fig. 7e).

**Table 5:** Physiological analysis of *TabZIP* overexpressing *Arabidopsis* plants showing increased tolerance to heat stress.

Parameters	Fv/Fm	YII	ETR	Membrane stability (%)	Proline content (mg/ml)
WT	0.55 ± 0.04	0.07 ± 0.02	1.73 ± 0.18	0.73 ± 0.008	0.22 ± 0.02
C 91.1	0.76 ± 0.01*	0.45 ± 0.02*	4.86 ± o.11*	0.69 ± 0.02	0.75 ± 0.01*
C 91.2	0.76 ± 0.02*	0.22 ± 0.02*	6.71 ± 0.38*	0.76 ± 0.007*	0.48 ± 0.04*
C 91.3	0.77 ± 0.002*	0.20 ± 0.02*	7.10 ± 0.33*	0.79 ± 0.01*	0.38 ± 0.02*
C91.4	0.77 ± 0.01*	0.17 ± 0.01*	5.67 ± 0.11*	0.70 ± 0.01	0.29 ± 0.05
C91.5	0.73 ± 0.01*	0.08 ± 0.01	7.50 ± 0.21*	0.71 ± 0.008	0.28 ± 0.02

*Represent P-value of ≤0.05; ±sign represent standard error.

### Role of *TabZIP* in oxidative stress

**Figure 8:**
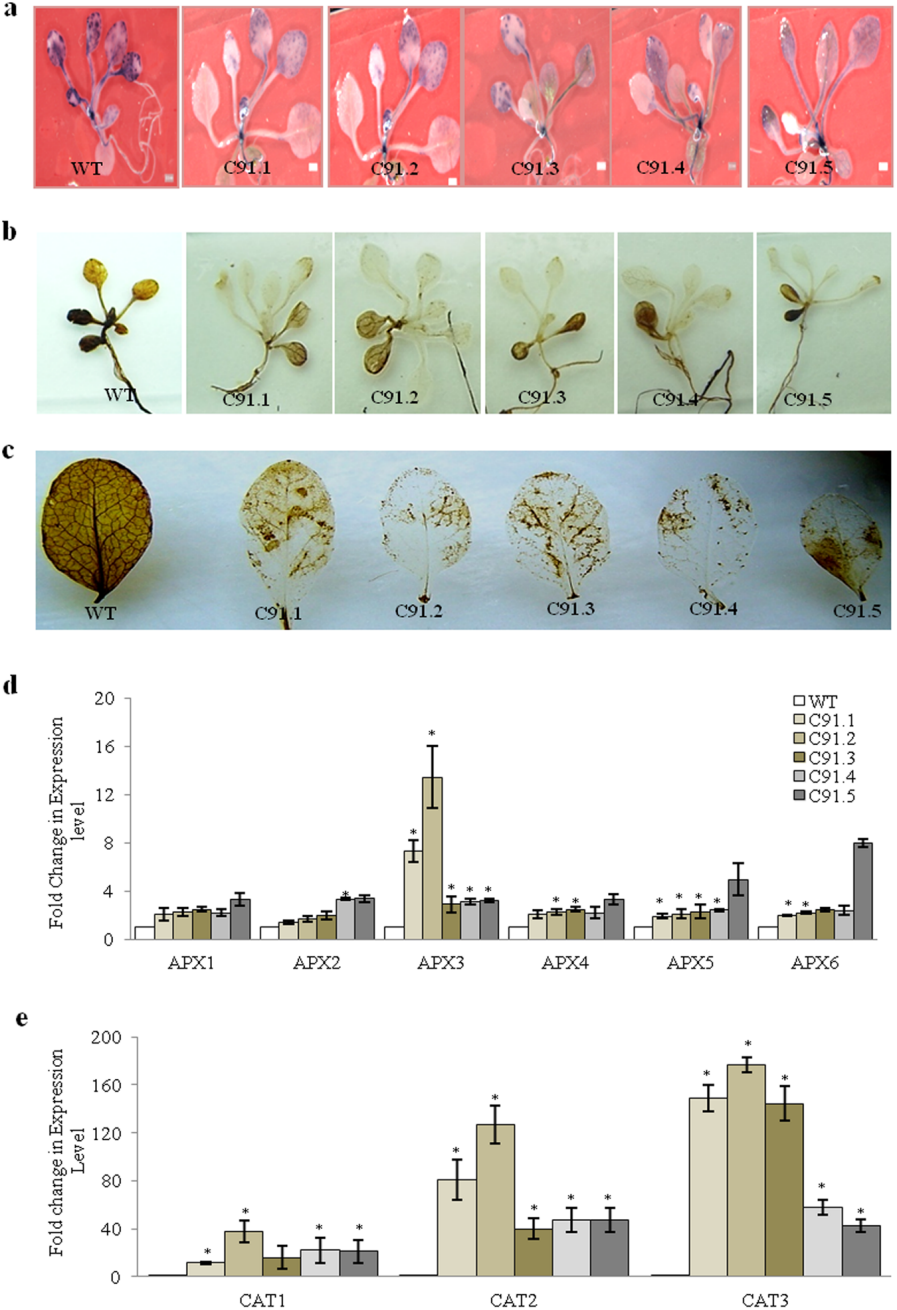
Oxidative stress response of *TabZIP* overexpressing plants. Oxidative stress response of wild type and *TabZIP* overexpressing *Arabidopsis* seedlings examined for comparative accumulation of ROS i.e. O_2_^−^ under heat stress i.e. 37 °C for two hrs by (**a)** NBT for O_2_^−^ species, (**b)** DAB for H_2_O_2_ species and (**c)** under oxidative stress treatment by methyl viologen i.e. 50 µM for four hrs by DAB staining. **(d)** Relative transcript abundance of APXs and (**e)** CATs in wild type and *TabZIP* overexpressing *Arabidopsis* plants. The expression level of wild type was normalized to 1.0. Results obtained are mean value ± standard deviations of minimum three independent experiments. *Asterick represents the significant difference i.e. Student’s t-test, P- value of ≤0.05.

Studies have reported that under unfavorable conditions like high temperature the plants show increased level of ROS concentration^40,41^. Thus, the effect of heat stress in terms of ROS accumulation was also measured in wild types and transgenics which were exposed to heat stress (37 °C) by both NBT (for O_2_^−^ species) as well as DAB (for H_2_O_2_ species) staining (Fig. 8a,b). Under heat stress conditions the transgenics showed less level of ROS accumulation than the wild types. In a similar way, level of ROS generated during oxidative stress was also visually quantified by DAB staining. For the oxidative stress assay, 21days old seedling of transgenics and wild types were given methyl vilogen treatment. It was found that under oxidative stress, hydrogen peroxide level were significantly low in transgenics as measured by DAB staining (Fig. 8c). It is well known that plants in response to heat stress produce antioxidant enzymes to scavenge the ROS produced during the stress. Hence the expression profiling of few oxidative stress related genes like *APX1*, *APX2*, *APX3*, *APX4*, *APX6*, *CAT1*, *CAT2* and *CAT3* was also undertaken to analyze the expression level of genes which results in lesser accumulation of ROS. This expression analysis showed increased levels of the marker genes in *TabZIIP* overexpression transgenics during heat stress (Fig. 8d,e).

## Discussions

In this previous report by Li *et al*. (2015), 187 bZIPs were identified^8^. However, in the current version of wheat genome 23 IDs given in the manuscript are obsolete now. Further in the same manuscript, ensembl ids for five bZIP genes were not provided. In this study, we have identified 191 bZIPs from wheat. The identified members are four more than in a previous report by Li *et al*.^8^ which is further supported by The Plant Transcription Factor Database i.e. TFDB (http://planttfdb.cbi.pku.edu.cn/index.php?spTae). The data used by the TFDB as well as by Li *et al*. 2015 was based on Ensembl version 35. However, present data is originated from latest Ensembl version 36.

bZIP transcription factors are known to participate in a wide range of developmental processes and stress response in plants. One of the greatest advantages of digitized data is the reusability and RNA-seq is one of the methods offering this opportunity to analyze the data in many contexts. In this study, a comparative analysis of expression profiles of *TabZIPs* in different stress conditions was done using RNA-seq data which revealed their differential expression under abiotic stress conditions. Further expression of one of the selected *TabZIP* gene (Traes_7AL_25850F96F.1) was found to be substantially induced by abiotic stress treatment like salt, heat, drought, cold stress and ABA treatment. Moreover, this response could be attributed to the presence of various *cis*-elements like DPBF1/2, Abscisic Acid Response Element and Low temperature Responsive Element found in its promoter region. Similarly, other *TabZIPs* too contained many stress responsive elements like DPBF1/2, Abscisic Acid Response Element and Low temperature Responsive Element, W box etc in their promoter regions. Besides the stress responsive elements they also contain hormone related *cis*-elements like ARF Binding Sites, Auxin Responsive Elements and Ethylene responsive elements. Drought responsive elements (DPBF1/2) were found in the 2 kb upstream region of various *TabZIPs* like Traes_7BL_625F55A12.1, Traes_6AL_362B70E63.1, TRAES3BF019000220FCD_t1, Traes_5BS_FF44610EF1.3, Traes_2BS_169BEF991.2, Traes_6AL_362B70E63.1 and Traes_6AL_E8CD2C02B.1 which further explains their high expression level under drought stress. These observations also corroborate with the previous findings where in wheat bZIP transcription factor like *WABI5*^29^ (under drought, low temperature and ABA), *TaABP1*^30^ and *TaABL1*^42^ (under salt, drought, low temperature and ABA), *TabZIP14-B*^43^ (under salt, low temperature, PEG and ABA) were reported to have induced expression under different abiotic stress treatments. *TabZIP60* was also found to participate in stress response^44^. Another bZIP from wheat *Wlip19*, homolog of *lip19* was also reported to be induced in response to low temperature, drought as well as by ABA treatment. Its expression was found to be highest in tolerant varieties in comparison to cold sensitive varieties^45^. Thus these results suggest that *TabZIP* participates in broad range of abiotic stresses.

Besides its role in heat and other abiotic stress response, *TabZIPs* including *TabZIP* (Traes_7AL_25850F96F.1) display temporal and varietal specific expression as evident from the RNA-seq and expression profiling studies. *TabZIPs* were found to be expressed in wide range of tissues ranging from seedling to reproductive tissues including stamens, pistil, anther, ovary, lemna, palea, awn, glume, endosperm and seeds at various developmental stages. This tissue specific expression of the bZIPs corroborates with the previous reports in wheat for genes like *TabABL1* (preferential expression in root, stem and leaves)^42^, *TaAREB3* (constitutive expression in leaf, stem, florets, anther, pistils and seeds with highest expression in roots)^46^, *TaABP1* (higher in leaves and stems than roots)^30^ and *TabZIP14-B* (expression in all tissues like stem, roots and spikes with maximum expression in leaves)^43^.

Besides the abiotic stress related and tissue specific expression under heat stress *TabZIPs* exhibit unique expression profile in different varieties of *Triticum aestivum*. Thus to conclude *TabZIP*s also respond in a varietal specific manner.

Trans-activation assays confirm that TabZIP has transactivation activity and its transactivation potential resides in C-terminal region as well as in the BRLZ domain. The presence of N-terminal was observed to be inhibitory for the transactivity of TabZIP.

The *Arabidopsis* transgenics overexpressing *TabZIP* showed increased tolerance to salt stress as transgenic seedlings exhibited healthier growth, higher photosynthetic efficiency, membrane stability and proline accumulation under salt stress conditions. They also showed better response during gradual salt stress treatments. The expression of various salt stress marker genes like *SOS1*, *SOS2* and *SOS3* were found to be comparatively more in overexpression transgenics in comparison to control plants. These observations substantiates with previous study where *Arabidopsis* transgenics overexpressing *TabZIP14-B* with increased expression of several stress marker genes like *RD20*, *RD29A*, *RAB18*, *GSTF6* and *COR47* showed increased tolerance to salt, low temperature and improved ABA sensitivity^45^. Similarly transgenic tobacco expressing *WABI5* showed enhanced tolerance to abiotic stresses like salt, freezing and osmotic stresses in the seedling stage in comparison to wild type^29^. In another monocot crop like rice the overexpression of *OsbZIP23* and *OsbZIP71* also resulted in improved tolerance to salt stress^13,19^. Thus these studies suggest that *TabZIP* positively mediates the expression of stress-responsive genes in transgenic plants under salt stress conditions, a role that has been shown to be common to many other stress-responsive bZIP transcription factors.

Apart from role in salt stress, *TabZIP* was also found to positively regulate drought stress response. The transgenics performed considerably well in comparison to wild type plants under both dehydration and drought stress. They showed better photosynthetic efficiency, proline content and membrane stability. They flowered earlier and produced more and larger siliques thereby resulting in greater yield under prolonged stress conditions. Moreover the expression of various drought stress marker genes like *ERD6*, *DREB1A*, *DREB2A*, *RD20*, *RD26*, *RD29A* and *RD29B* were also found to be up-regulated in transgenics. These results are similar to the previous study by Li *et al*., 2016 where *Arabidopsis* transgenics overexpressing *TaBZIP174* showed enhanced tolerance to drought stress as they displayed longer root, higher survival rate, more proline content as well as chlorophyll content and also displayed stable osmotic potential^47^. Moreover, these transgenics also showed increased expression level of various stress marker genes like *RAB18*, *RD29A*, *RD29B*, *DREB2A*, *COR47* and *COR15A*. Similarly *TaAREB3* overexpression resulted in enhanced tolerance to drought, low temperature and improved sensitivity to ABA as well as increased expression of *RD29A*, *RD29B*, *COR47* and *COR15A* in *Arabidopsis* transgenics^46^. Another study on *TaABP1* in tobacco has shown increased tolerance in transgenics under drought stress^30^. In rice, *OsbZIP23* is known to act as positive regulator and genes encoding dehydrin or LEA proteins were found to be upregulated in the overexpression rice transgenics^13^. Moreover studies of few other *OsbZIP* genes in rice, like *OsbZIP16*^16^ and *OsbZIP71*^19^ have reported them as positive regulators of drought and osmotic stress. The expression of various drought stress marker genes was found to be higher in these transgenics lines. Thus, together these results demonstrate that *TabZIP* is a positive regulator of drought stress as it regulates the expression of various stress responsive genes in transgenic plants.

In addition to salt and drought stress, present work elucidates conclusively that overexpression of *TabZIP* resulted in increased thermal stress tolerance in transgenic *Arabidopsis* lines. The transgenics showed better performance w.r.t to their post stress recovery, growth and yield. They also exhibited higher Fv/FM, ETR, YII, proline accumulation, chlorophyll retention and membrane stability. The size and number of siliques as well as total yield per plant was more and these could be one of the possible reasons for the overall increased yield of the transgenic plants under heat stress. Further the expression of heat stress associated genes like *HSF* and *HSP101* was found to be significantly high in transgenics when compared to wild types. ROS accumulation, one of the detrimental effects of heat stress, was also found to be considerably less in transgenics than the wild type under high temperature and oxidative stress. Lesser accumulation of ROS could be due to high activity of oxidative stress marker genes including *APXs* and *CATs* whose expression was observed to be higher in transgenics. Thus these results suggest that *TabZIP* provides thermotolerance by enhancing the accumulation of the osmolyte and at the same time may be positively regulating the expression of heat and oxidative stress associated genes so as to minimize the production of ROS to overcome its detrimental effects. These observations are in agreement with the previously published report where the expression of *OsbZIP46* was found to be strongly induced by heat and hydrogen peroxide treatment^17^.

Therefore, it can be concluded from the above experiments that *TabZIP* acts as a positive regulator in various stress response like salt, drought, heat and oxidative stress. This is further supported by wide range of various abiotic stress related cis-acting elements present in the promoter region of these genes. Moreover, this dramatic increase in tolerance of the transgenic plants to various stress conditions is mediated via regulation of various stress associated marker genes at the transcriptional level. Changes in the metabolite level and overall improvement of the various physiological responses like photosynthetic efficiency, membrane stability and chlorophyll content is also observed and this is reflective of the better adaptability of the *TabZIP* overexpression transgenics under adverse abiotic conditions.

To summarize, *TabZIP* positively regulates salinity, drought, heat and oxidative stress response. Thus the above analysis helped in deciphering the role of *TabZIP* in heat and abiotic stress responses as well as in plant growth and development. The fact that overexpression of *TabZIP* dramatically resulted in increased tolerance to salinity, drought and heat and oxidative stress of transgenic *Arabidopsis* reflects on the application of deploying this gene in crop plants for abiotic stress tolerance in general and for heat stress tolerance in particular.

## Methods

### Identification of bZIP gene family members and phylogenetic analysis

Blast search and hmm search methods were employed to identify bZIP encoding members in wheat. Briefly put, two hmm profiles corresponding to bZIPs with accession numbers PF00170.16 and PF07716.10 were fetched from PfamA.hmm file. PfamA.hmm file is a manually curated database for the hmm profiles of over 16,000 different gene families. To fetch the hmm profiles hmmfetch software of hmmsuit HMMER version 3.1b2 for linux platform was used. Fetched hmm profiles were used to find the predicted proteins of wheat from Ensembl FTP^48^ (ftp://ftp.ensemblgenomes.org/pub/release29/plants/fasta/triticumaestivum/pep/Triticum_aestivum.IWGSC1.0+popseq. 29.pep.all.fa.gz). HMM being a sensitive method will also catch splice variants too. To overcome this, manual curation was applied to resolve the issue of splice variants after HMM searches. In the annotation process, splice variants are named as gene names and an added numerical to report splice variants. Further, *Arabidopsis* and rice proteins having these pfam domains were downloaded from TAIR (https://www.Arabidopsis.org/tools/bulk/sequences/index.jsp) and RGAP (http://rice.plantbiology. msu.edu/) databases respectively. Rice and *Arabidopsis* bZIPs proteins were Blast searched against the above wheat protein sequences. Proteins thus identified from hmm and BLAST searches were further searched for presence of conserved bZIP domain using NCBI-CDD server (http://www.ncbi.nlm.nih.gov/Structure/cdd/cdd.shtml ^49^.

Domains of bZIPs from these proteins were fetched using a custom written perl script employing samtools^50^. Using hmmbuild in HMMER suit, a new hmm profile was built and wheat proteins were searched. This exercise was repeated till no new members could be identified.

To conduct phylogenetic analysis, bZIP domains of wheat, rice and *Arabidopsis* proteins were used. These domain sequences were aligned in Clustal X version 2.1 and based on this alignment;, a neighbour joining (NJ) phylogenetic tree has been constructed in MEGA version 7.0. Bootstrap confidence level for this tree was assessed with 1000 replications and the tree was edited for aesthetic changes in Fig tree version 1.4.2^51^. Another NJ tree was made by using the alignment file generated from whole protein sequences of wheat in order to understand the evolution of its members.

### Structure of *TabZIP* genes and conserved motifs in TabZIP proteins

Data comprising gene features were fetched from the GFF3 file provided from Ensembl wheat database^52^ (ftp://ftp.ensemblgenomes.org/pub/release-29/plants/gff3/triticumaestivum/). The coordinates for UTR, CDS etc. were fetched and converted in bed format followed by gene structure diagram that was generated using GSDS (Gene Structure Display Server) version 2.0^53^. MEME motif search was performed at http://meme-suite.org/ to identify motifs present in these proteins with parameters including identification of 15 motifs with any number of repetitions with optimal motif length of 8 to 50 residues^54^. Conserved motifs were annotated using iprscan tool (http://www.ebi.ac.uk/Tools/pfa/iprscan/).

### Gene ontology and expression profile of bZIPs in different varieties, growth and developmental stages

Gene ontology terms were fetched from Gramene FTP^55^ (ftp://ftp.gramene.org/pub/gramene/CURRENT_RELEASE/data/ontology/go/go_ensembl_triticumaestivum.gaf). Gene ontology (GO) terms for biological process, molecular function and cellular components were analyzed for their enrichment. A hypergeometric distribution test (p value ≤ 0.05) with false discovery rate estimation using Benjamini-Hochberg procedure in Bingo plugin^56^ in Cytoscape version 3.02^57^ was performed. To analyze the expression pattern of these bZIPs across the developmental stages, stresses and varieties, normalized data in form of TPM values (Transcripts per million) from Wheat Expression server^32^ (http://wheat-expression.com/) for wheat were downloaded. Heat maps depicting the expression pattern in 11 developmental and stress stages and 12 varieties were plotted in R.

### Plant material, growth conditions and stress treatments

Five different varieties of *Triticum aestivum* L. c.v. PBW343, HD2329, CPAN1676, K7903, C306 and *Arabidopsis thaliana* (ecotype Col0) were used for the experiment. For wheat seedling studies, seeds were sown in plastic trays and maintained in growth chamber (Conviron®, Canada) at 20 ± 1 °C; 16 hours light: 8 hours dark photoperiodic cycle. While *Arabidopsis* seedlings were grown on MS media at 22 ± 1 °C and 16 hours light: 8 hours dark cycle. For other plant development stages, the plants were grown in soilrite for *Arabidopsis* and in soil for wheat. For heat stress experiments, ten-days seedlings of wheat variety PBW343 were given stress in growth chambers set at required temperatures i.e. 37 °C and 42 °C for 2 hrs and 4 hrs. After heat stress treatment, for recovery the seedlings were transferred back to the growth chamber for 2 hrs and 4 hrs respectively. For other abiotic stress treatment, ten-days old PBW343 seedlings were exposed to salt stress (150 mM NaCl for two hrs), dehydration (2% mannitol for two hrs), cold (4 °C for 24 hrs) and ABA (10 µm for two hrs). For developing grains, spikes at 3, 5, 7, 10 and 20 DAA from PBW343 were subjected to 37 °C for two hrs. Sample of various tissues i.e. awn, glume, lemma, palea, anther and ovary was collected from both control and stress treated plants at 37 °C. *Arabidopsis* seedling were given salt stress by supplementing MS medium containing 150 mM NaCl, and then to further monitor the overall growth 14-day-old wild types and transgenics (normal plants grown under culture room conditions in soilrite pots) were also given gradual salt stress treatment i.e. watered with 150 mM NaCl solution for four days followed by 200 mM NaCl for another four days, and then 300 mM NaCl for sixteen days. For dehydration stress experiment, wild type and transgenic seeds were grown on MS medium supplemented with 4% PEG and further evaluated. For drought experiments, 22-day-old seedlings were grown in soilrite and then subjected to drought stress for 14 days and then rewatered for 5 days. To monitor overall growth and yield, almost one month old plants were given drought stress for one month. For heat stress studies, 7-day-old control and transgenic seedlings were given heat stress at 42 °C (for two hrs) and followed by recovery at 22 °C. For oxidative stress, 14-days old plants were treated for 4 hrs with 50 µM methyl vilogen.

### RNA isolation and expression analysis by real-time PCR

Total RNA from all the samples were isolated using Tri Reagent® (Sigma Aldrich, Germany). RNAeasy Plant Mini Kit (Part No. 74903; Qiagen) was used to clean up the isolated samples followed by DNase treatment on the same columns. Quality assessment was done by spectrophotometer (NanoVue; GE Healthcare) and RNA samples were checked on 1% denaturing agarose gels. 2.0 µg of total RNA from each samples were taken for cDNA preparation. High capacity cDNA archive kit (P/N 4322171; Applied Biosystems) was used to prepare cDNA samples as per the manufacturer’s protocol. The primers were designed in Primer Express version 2.0 (PE Applied Biosystems, USA) following default parameters. The real-time PCR reactions were carried out in ABI Prism 7000 sequence detection system (Applied Biosystems, USA) under the following set of conditions including melt curve and dissociation curve: 95 °C for 10 min, 95 °C for 30 sec, 60 °C for 1 min, 72 °C for 1 sec; 95 °C for 1 min, 60 °C for 30 sec, 95 °C for 30 sec and 40 cycles. Average of two biological replicates with three technical replicates each was plotted. The data was normalized using *actin* gene as internal control. The relative expression was estimated using 2-ΔΔCT method. Primers used are listed in Supplementary Table S6.

To analyze the global expression pattern of wheat bZIPs, ExVIP server based expression values were used. ExVIP server is hosted at www.wheat-expression.com. It harbors the normalized expression values from publicaly available wheat RNAseq experiments^32^. TPM values for corresponding genes in different developmental stages and stressed tissues were fetched and the same was used to generate the heatmap in R.

### Raising of *Arabidopsis* transgenics overexpressing *TabZIP* (Traes_7AL_25850F96F.1)

*TabZIP* ORF was PCR amplified from wheat variety CPAN1676 and initially cloned into pGEM-T Easy vector. For over-expression studies, the ORF was PCR amplified using pGEM-T Easy:*TabZIP* plasmid taking Topo-*TabZIP*-Forward and Topo-*TabZIP*-Reverse primers (Supplementary Table S6) and cloned into pENTR/D-TOPO (Invitrogen) vector. This construct was further inserted into pMDC32 vector using LR-clonase based reaction according to manufacturer guidelines. *Arabidopsis thaliana* Col-0 were transformed by floral dip method^58^. For analysis T3 homozygous seeds and five transgenic lines were used. The data presented here represents at least 3 replicates.

### Transactivation assay of *TabZIP* (Traes_7AL_25850F96F.1)

For transactivation activity, complete CDS of *TabZIP* was cloned in pGBKT7 vector using primers having *Eco*RI and *Bam*HI restriction sites respectively in the 5′ and 3′ ends (Supplementary Table S6). Various deletion constructs were prepared in order to delineate the function of its various domains in conferring transactivation potential using primers given in Supplementary Table S6. Schematic representation of full length and deletions of TabZIP protein for transactivation and interaction (Y2H) assay (Supplementary Fig. S10). Yeast strain AH109 was used for Y1H assay and trans-activation assay was performed on both the SD-H and SD-HW media.

### Chlorophyll fluorescence measurements

Chlorophyll fluorescence was estimated using Krause and Weis (1991) protocol^59^. Chlorophyll fluorescence emission was measured from upper surface of the leaf using Pulse Amplitude Modulation fluorometer (Junior-PAM chlorophyll fluorometer, H. Walz, Germany). Leaves from control and treated seedlings were initially kept for 20 min in dark before measuring the fluorescence induction. Parameters like maximum photosynthetic efficiency (Fv/Fm), effective photosynthetic efficiency (YII) and Electron Transport Rate (ETR) were measured using at least 10 plants per line from rosette leaves of both control and transgenics seedlings.

### Total chlorophyll estimation

Chlorophyll amount was estimated according to Hiscox and Israelstam^60^. For chlorophyll estimation, 0.05 g of leaf samples from control and stressed plants in five different replicates were taken and incubated in DMSO (5 ml) and kept in dark, at 65 °C for 4 hrs. Absorbance of samples were measured at wavelengths 645 and 663 nm using spectrophotometer (Beckman DUTM 640B). Chlorophyll content was calculated using following formula.

$${\rm{Chla}}=[(12:3{\rm{A}}663-0:86{\rm{A}}645){\rm{xV}}]/{\rm{X}}\ast 1000\ast {\rm{W}}$$

$${\rm{Chlb}}=[(19:3{\rm{A}}645-3:6{\rm{A}}663){\rm{xV}}]/{\rm{X}}\ast 1000\ast {\rm{W}}$$

where, V = DMSO volume (ml), X = path length (1 cm) and W = fresh weight (g).

### Membrane stability estimation

The membrane stability was calculated using the protocol by Bajji *et al*.^61^. For cell membrane stability, the seedlings were initially submerged in distilled water for 30 min maintained at 30 °C. Measurement of electrical conductivity C1 was done follwed by autoclaving for 15 min and then electrical conductivity C2 was measured. Calculation of membrane stability was done using the given formula.

$${\rm{Membrane}}\,{\rm{Stability}}\,{\rm{Index}}=[1-({\rm{C}}1-{\rm{C}}2)].$$

### Estimation of Proline content

Total amount of proline was measured as per the protocol by Bates *et al*.^62^. To calculate the proline content, approximately 100 mg of leaf tissue was ground in 3% sulphosalicyclic acid (1 ml), centrifuged for 15 min at 15000 rpm. To the obtained supernatant glacial acetic and ninhydrin (400 µl each) was added. The samples were then incubated for 1 hr at 100 °C. The reaction was then inhibited by keeping the samples in ice followed by addition of toluene (800 µl) and mixed by vigorous vortexing. Absorbance was measured at 520 nm wavelength using the supernatant of the samples using UV-visible spectrophotometer (model U-2810 Spectrophotometer). The proline content of the test samples were measured using the following formula.

$$\mu \text{moles}\,{\rm{of}}\,{\rm{proline}}/{\rm{gm}}\,{\rm{fresh}}\,{\rm{Wt}}=(\mu g\,{\rm{protein}}/{\rm{ml}}\times {\rm{ml}}\,{\rm{toluene}}\,5)/115.5\,\mu g/\mu \text{moles}\times {\rm{gm}}\,{\rm{sample}}$$

### ROS estimation

ROS (reactive oxygen species) level was measured by both NBT (Nitro blue tetrazolium chloride) and DAB (3,3′-Diaminobenzidine) staining. For NBT staining 10-days old seedlings (control and test samples) of *Arabidopsis* wild type and transgenics were incubated (for staining) in NBT (2 mM NBT powder, 20 mM phosphate buffer) for overnight^63^. While for DAB staining the seedlings were incubated in DAB (0.05% v/v Tween twenty, 100 mM phosphate buffer, 200 mM Na_2_PO_4_, pH 3.0) for overnight^64^. Next day washing was done using MQ water followed by removal of chlorophyll by keeping the samples for 2 hrs in bleaching solution (acetic acid, glycerol and ethanol in 1:1:3 ratio).

### Statistical analysis

Statistical analysis was done by calculating the average or mean value and standard error for all the replicates. Student’ t-test was calculated to find the significant difference between wild type and transgenic lines. P-value of ≤0.05 was taken as significant and is represented by *. Data presented in this study represents three biological replicates with at least 5–10 seedlings per experiment.

## Supplementary information

Supplementary Information

Supplementary Table S1

Supplementary Table S2

Supplementary Table S3

## Data Availability

The data generated during the current study are incorporated in this manuscript and supplied in form of supplementary files.
