# Supplementary material for: Genome-wide Analysis of bZIP Transcription Factors in wheat and Functional Characterization of a TabZIP under Abiotic Stress
Source: Sci Rep. 2019 Mar 14;9:4608. doi: 10.1038/s41598-019-40659-7 (PMC6418127; doi:10.1038/s41598-019-40659-7)
Supplement: Supplementary file 1 — Supplementary Information [file 41598_2019_40659_MOESM1_ESM.pdf]

Supplementary Information

Genome-wide Analysis of bZIP Transcription Factors in wheat and Functional Characterization of a *TabZIP* under Abiotic Stress

Preeti Agarwal[1], Vinay Kumar Baranwal[2] & Paramjit Khurana[1*]

Affiliations

[1]Department of Plant Molecular Biology, University of Delhi South Campus, Benito Juarez Road, New Delhi, 110021, India

[2]Department of Botany, Swami Devanand Post Graduate College, Devashram Marg, Lar, Deoria, 274502, India

[*]**Corresponding Author**

Paramjit Khurana

[1 & 2] correspond to Affiliations

E-mail address, telephone and fax of corresponding author

Corresponding Author email: param@genomeindia.org

Phone: +91-11-24115096

Fax: +91-11-24115095

PA email: preetiagarwal86du@gmail.com

VKB email: vinaydu@gmail.com

Supplementary Figures

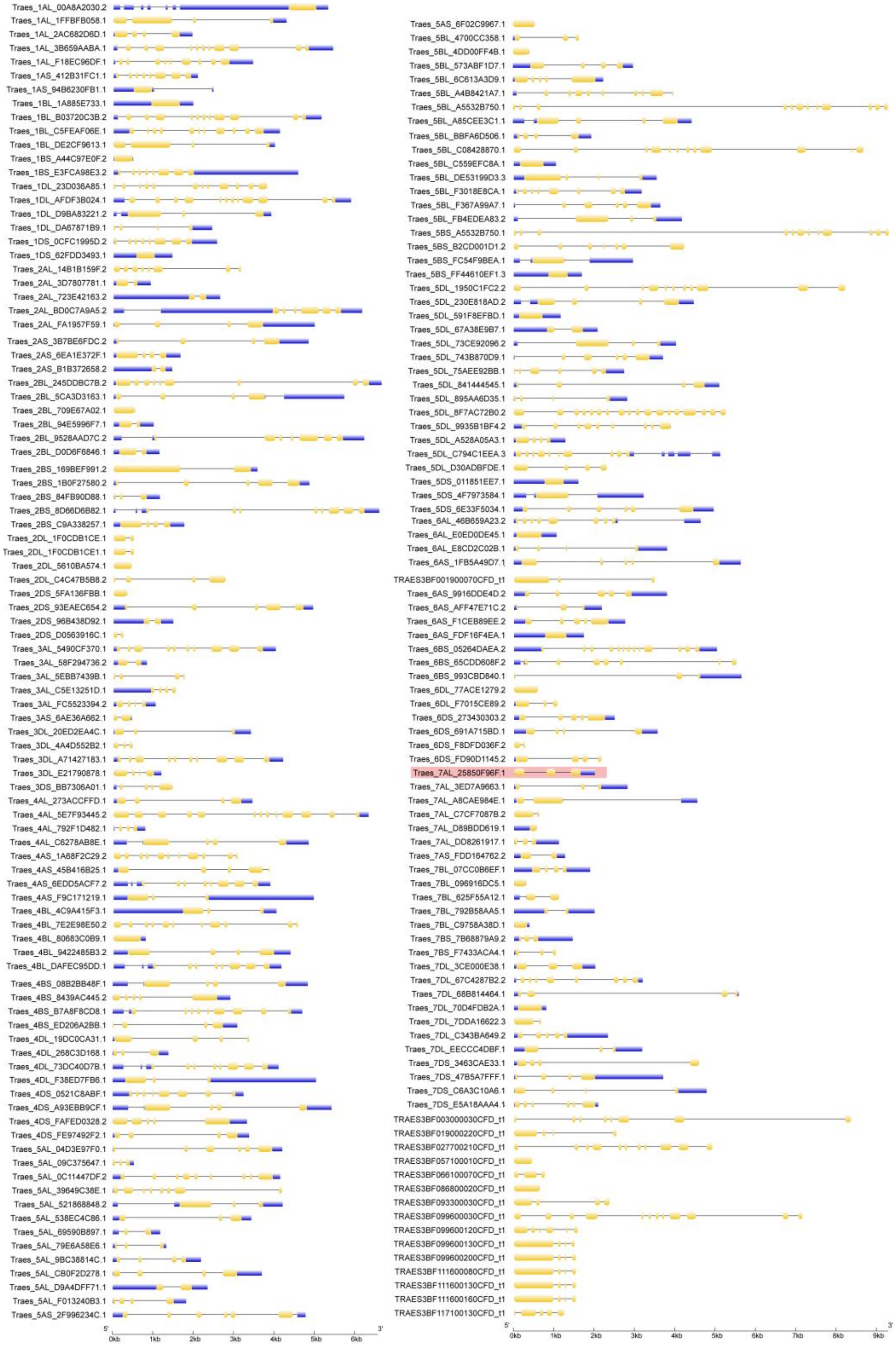

Supplementary Figure S1. Gene structures diagram depicting the distribution of exons and introns in the identified bZIP components of *Triticum aestivum* cv. Chinese spring. Exons are represented using solid boxes while introns are depicted using solid lines. UTRs are shown using blue boxes.

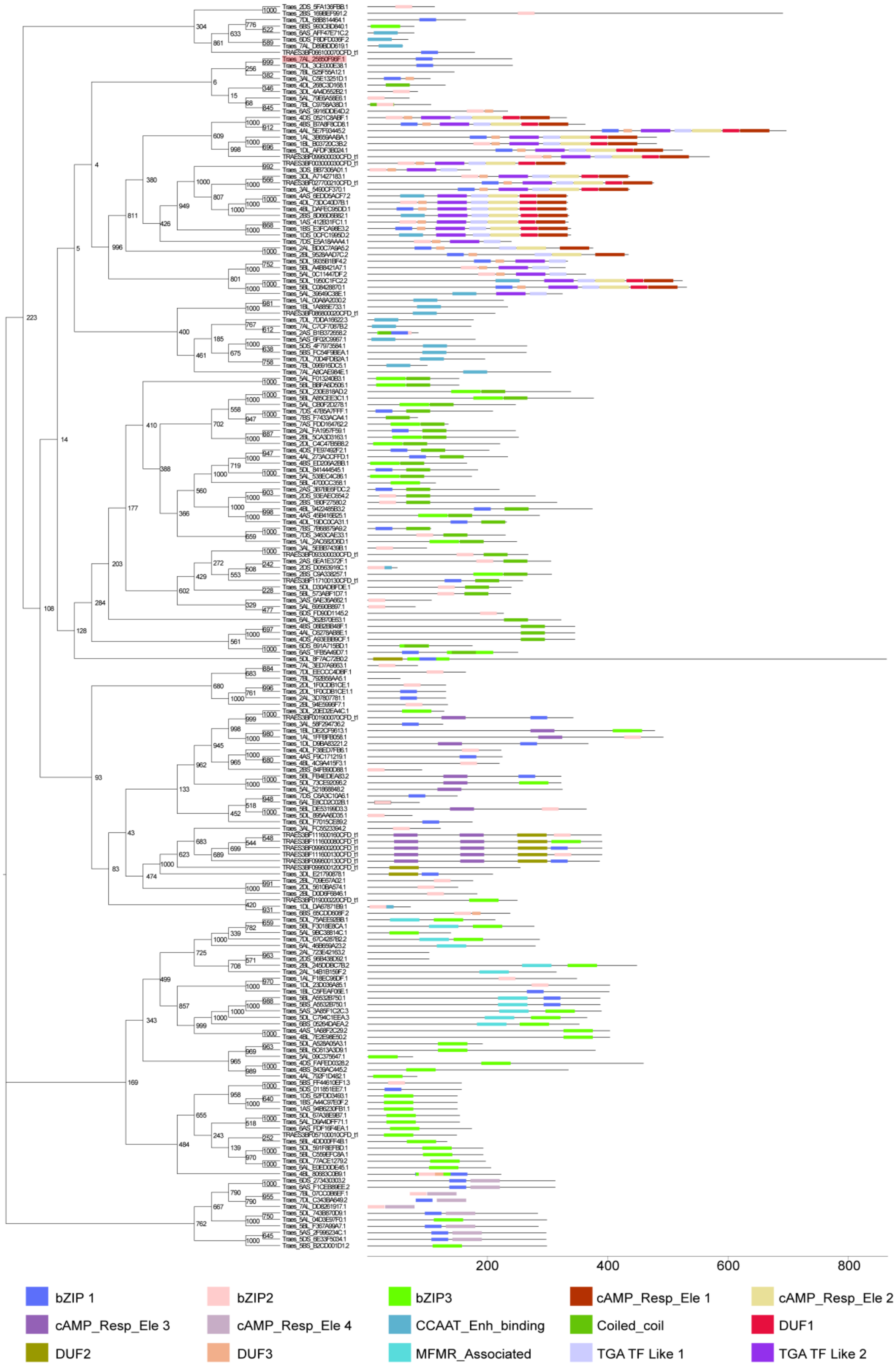

Supplementary Figure S2. Phylogenetic distribution of wheat bZIPs and presence of domains in their protein: Numbers on the nodes of the cladogram depicts the bootstrap replication values. Conserved domains identified in these proteins are shown using solid boxes of different colors (See text for details).

```
Traes_7BL_625F55A12.1      ------MRNRDSAMKSRERKKS-----
Traes_7AL_25850F96F.1      --KMRQMRNRDSAMKSRERKKS-----
Traes_7DL_3CE000E38.1      --KRRQMRNRDSAMKSRERKKS-----
Traes_6DS_F8DFD036F.2      -----LLRNRVSAQQARERKKA-----
Traes_6AS_AFF47E71C.2      --RAGLLRNRVSAQQARERKKA-----
Traes_7AL_D89BDD619.1      --QSRLLRNRVSAQQARERKKAYMG--
Traes_6BS_993CBD840.1      --LKRLLRNRVSAQQARERKKA-----
Traes_7DL_68B814464.1      --LKRLLRNRVSAQQARERKKA-----
TRAES3BF066100070CFD_t1    --LKRLLRNRVSAQQARERKKA-----
Traes_2DS_5FA136FBB.1      --RARLVRNRESAHLSRQRKKQ-----
Traes_2BS_169BEF991.2      --RARLVRNRESAHLSRQRKKQ-----
Traes_5AL_521868848.2      --QKRMIKNRESAARSRARKQG-----
Traes_1AL_1FFBFB058.1      --QKRMIKNRESAARSRARKQA-----
Traes_1BL_DE2CF9613.1      --QKRMIKNRESAARSRARKQA-----
Traes_1DL_D9BA83221.2      --QKRMIKNRESAARSRARKQA-----
Traes_5DL_73CE92096.2      --QKRMIKNRESAARSRARKQA-----
Traes_5BL_FB4EDEA83.2      --QKRMIKNRESAARSRARKQA-----
Traes_5DL_895AA6D35.1      -----MIKNRESAARSRARKQA-----
Traes_5AL_79E6A58E6.1      -----MIKNRESAARSRARKQA-----
Traes_6AL_E8CD2C02B.1      --QRRMIKNRESAARSRQRKQS-----
Traes_6DL_F7015CE89.2      --QRRMIKNRESAARSRQRKQS-----
Traes_7DS_C6A3C10A6.1      --QRRMIKNRESAARSRQRKQA-----
Traes_2DL_5610BA574.1      --QRRMIKNRESAARSRARKQA-----
Traes_2BL_709E67A02.1      --QRRMVKNRESAARSRARKQA-----
Traes_2BL_D0D6F6846.1      --QRRMIKNRESAARSRARKQA-----
Traes_5BL_DE53199D3.3      --QRRMIKNRESAARSRARKQA-----
Traes_2BS_84FB90D88.1      -----MIKNRESAARSRARKQA-----
TRAES3BF099600200CFD_t1    --HRRMIKNGESAARSRARKQA-----
TRAES3BF111600080CFD_t1    --HRRMIKNGESAARSRARKQA-----
Traes_3AL_FC5523394.2      --HRRMIKNRESAARSRARKQA-----
TRAES3BF111600160CFD_t1    --HRRMIKNRESAARSRARKQA-----
TRAES3BF099600130CFD_t1    --HRRMIKNRESAARSRARKQA-----
TRAES3BF111600130CFD_t1    --HRRMIKNRESAARSRARKQA-----
TRAES3BF019000220CFD_t1    --HKRMIKNRESAARSRERKQA-----
Traes_3DL_E21790878.1      --HHRMIKNRESAGQSRARKQA-----
Traes_3AL_58F294736.2      --VSGDVPNKFVERRQKRMIKN-----
TRAES3BF001900070CFD_t1    --VSGDVPNKFVERRQKRMIKN-----
Traes_3DL_20ED2EA4C.1      -------PNKFVERRQKRMIKNRESAA
Traes_7AL_3ED7A9663.1      --TIRMMRNRESALRSRARKRA-----
Traes_7DL_EECCC4DBF.1      --TIRMMRNRESALRSRARKRA-----
Traes_2DL_1F0CDB1CE.1      --SVRAMKNRESALRSRARKRA-----
Traes_2DL_1F0CDB1CE1.1     --SVRAMKNRESALRSRARKRA-----
Traes_2AL_3D7807781.1      --SVRAMKNRESALRSRARKRA-----
Traes_2BL_94E5996F7.1      --SIRAMKNRESALRSRARKRA-----
Traes_7BL_07CC0B6EF.1      --IRRMVSNRESARRSRRRKHA-----
Traes_7DL_C343BA649.2      --IRRMVSNRESARRSRRRKHA-----
Traes_7AL_DD8261917.1      -----MVSNRESARRSRRRKHA-----
Traes_1AS_94B6230FB1.1     --RKRMLSNRESARRSRARKQQ-----
Traes_1DS_62FDD3493.1      --RKRMLSNRESARRSRARKQQ-----
Traes_1BS_A44C97E0F.2      --RKRMLSNRESARRSRARKQQ-----
Traes_5DL_591F8EFBD.1      --RRRMVSNRESARRSRMRKQR-----
Traes_5BL_C559EFC8A.1      --RRRMVSNRESARRSRMRKQR-----
Traes_6AL_E0ED0DE45.1      --RRRMVSNRESARRSRMRKQK-----
```

```
Traes_6DL_77ACE1279.2      --RRRMVSNRESARRSRMRKQK-----
Traes_6AS_FDF16F4EA.1      --AKRMLSNRESARRSRMRKQR-----
Traes_5BS_B2CD001D1.2      --VKRMLSNRESARRSRKRKQA-----
TRAES3BF057100010CFD_t1    --RKRKESNRLSAQRSRARKLL-----
Traes_4BL_80683C0B9.1      --RKRKTSNRLSAQRSRMKRQQ-----
Traes_5BL_4DD00FF4B.1      --QRRKVSNRLSAQRSRARKQQ-----
Traes_5BS_FF44610EF1.3     --EKRRLSNRESARRSRLRKQQ-----
Traes_5DS_011851EE7.1      --EKRRLSNRESARRSRLRKQQ-----
Traes_5AL_9BC38814C.1      --ERRKQSNRESARRSRLRKQQ-----
Traes_5DL_75AEE92BB.1      --ERRKQSNRESARRSRLRKQQ-----
Traes_7DL_67C4287B2.2      --ERRKQSNRESARRSRLRKQQ-----
Traes_5BL_F3018E8CA.1      --ERRKQSNRESARRSRLRKQQ-----
Traes_2BL_245DDBC7B.2      --ERRKQSNRESARRSRLRKQQ-----
Traes_2AL_14B1B159F.2      --ERRKQSNRESARRSRLRKQV-----
Traes_5DL_67A38E9B7.1      --KRRKESNRESARRSRVRKQQ-----
Traes_5AL_D9A4DFF71.1      --KRRKESNRESARRSRVRKQQ-----
Traes_5BL_F367A99A7.1      --RRRKQSNRESARRSRSRKAA-----
Traes_5DL_743B870D9.1      --RRRKQSNRESARRSRSRKAA-----
Traes_5AL_04D3E97F0.1      --RRRKQSNRESARRSRSRKAA-----
Traes_2DS_D0563916C.1      -----ILANRQSAQRSRVRKLQ-----
Traes_5AL_69590B897.1      ----MILANRQSAQRSRVRKLQ-----
Traes_3AL_5EBB7439B.1      --VKRILANRQSAQRSRVRKLQ-----
Traes_5BL_573ABF1D7.1      --VKRILANRQSAQRSRVRKLQ-----
Traes_5DL_D30ADBFDE.1      --VKRILANRQSAQRSRVRKLQ-----
TRAES3BF093300030CFD_t1    --VKRILANRQSAQRSRVRKLQ-----
Traes_2AS_6EA1E372F.1      --VKRILANRQSAQRSRVRKLQ-----
Traes_2BS_C9A338257.1      --VKRILANRQSAQRSRVRKLQ-----
TRAES3BF117100130CFD_t1    --VKRIIANRQSAQRSRVRKLQ-----
Traes_3AS_6AE36A662.1      ----RILANRQSAQRSRVRKLH-----
Traes_4BS_ED206A2BB.1      --AKRILANRQSAARSKERKIK-----
Traes_5DL_841444545.1      --AKRILANRQSAARSKERKIK-----
Traes_5BL_4700CC358.1      --AKRILANRQSAARSKERKIK-----
Traes_4DS_FE97492F2.1      --AKRILANRQSAARSKERKIK-----
Traes_4AL_273ACCFFD.1      --AKRILANRQSAARSKERKIK-----
Traes_5AL_538EC4C86.1      --LCRILANRQSAARSKERKIK-----
Traes_7DS_47B5A7FFF.1      --AKRIWANRQSAARSKERKMR-----
Traes_5AL_CB0F2D278.1      --AKRIWANRQSAARSKERKMR-----
Traes_5DL_230E818AD.2      --AKRIWANRQSAARSKERKMR-----
Traes_5BL_A85CEE3C1.1      --AKRIWANRQSAARSKERKMR-----
Traes_2AL_FA1957F59.1      --AKRIMANRQSAARSKERKMR-----
Traes_2BL_5CA3D3163.1      --AKRIMANRQSAARSKERKMR-----
Traes_7AS_FDD164762.2      --CDRIWANRQSAARSKERKMR-----
Traes_2DL_C4C47B5B8.2      --NLVIMANRQSAARSKERKMR-----
Traes_7BS_7B68879A9.2      --VKRILANRQSAARSKERKMR-----
Traes_1AL_2AC682D6D.1      --VKRVLANRQSAARSKERRMR-----
Traes_2AS_3B7BE6FDC.2      --AKRILANRQSAARSKERKAR-----
Traes_2DS_93EAEC654.2      --AKRILANRQSAARSKERKAR-----
Traes_2BS_1B0F27580.2      --AKRILANRQSAARSKERKAR-----
Traes_4AS_45B416B25.1      --AKRILANRQSAARSKERKAR-----
Traes_4DL_19DC0CA31.1      --AKRILANRQSAARSKERKAR-----
Traes_4BL_9422485B3.2      --AKRILANRQSAARSKERKAR-----
Traes_5AL_F013240B3.1      --VRRILNNRLSAAKSKERKAK-----
Traes_5BL_BBFA6D506.1      --VRRILNNRLSAAKSKERKAK-----
```

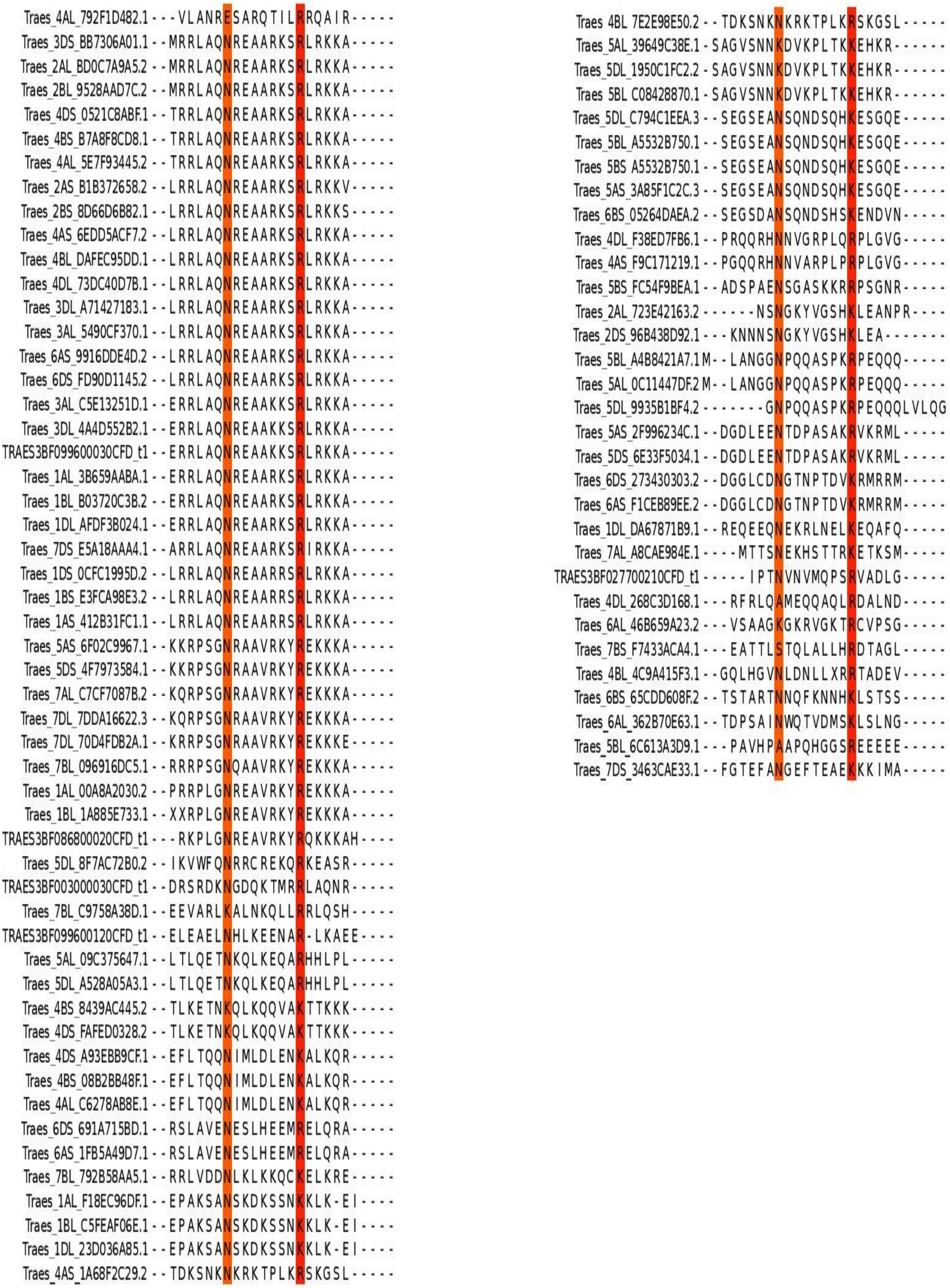

Supplementary Figure S3. Multiple sequence alignment of identified proteins with conserved 'N-x7-R/K' motif highlighted in red

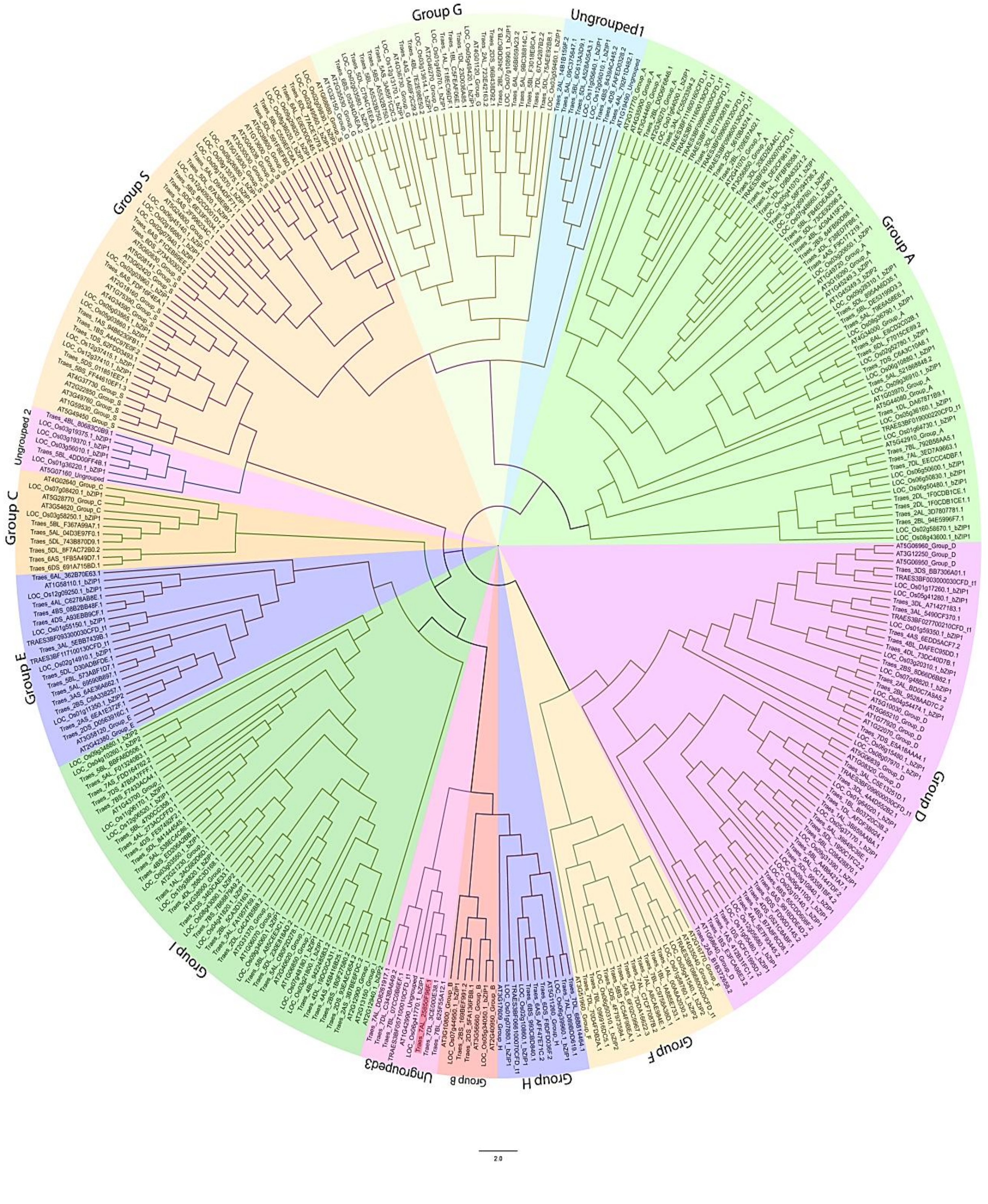

Supplementary Figure S4. Unrooted neighbor joining phylogenetic tree displaying the distribution of wheat, rice and *Arabidopsis* bZIPs into their respective clades. Different clades are shown using contrasting coloured backgrounds.

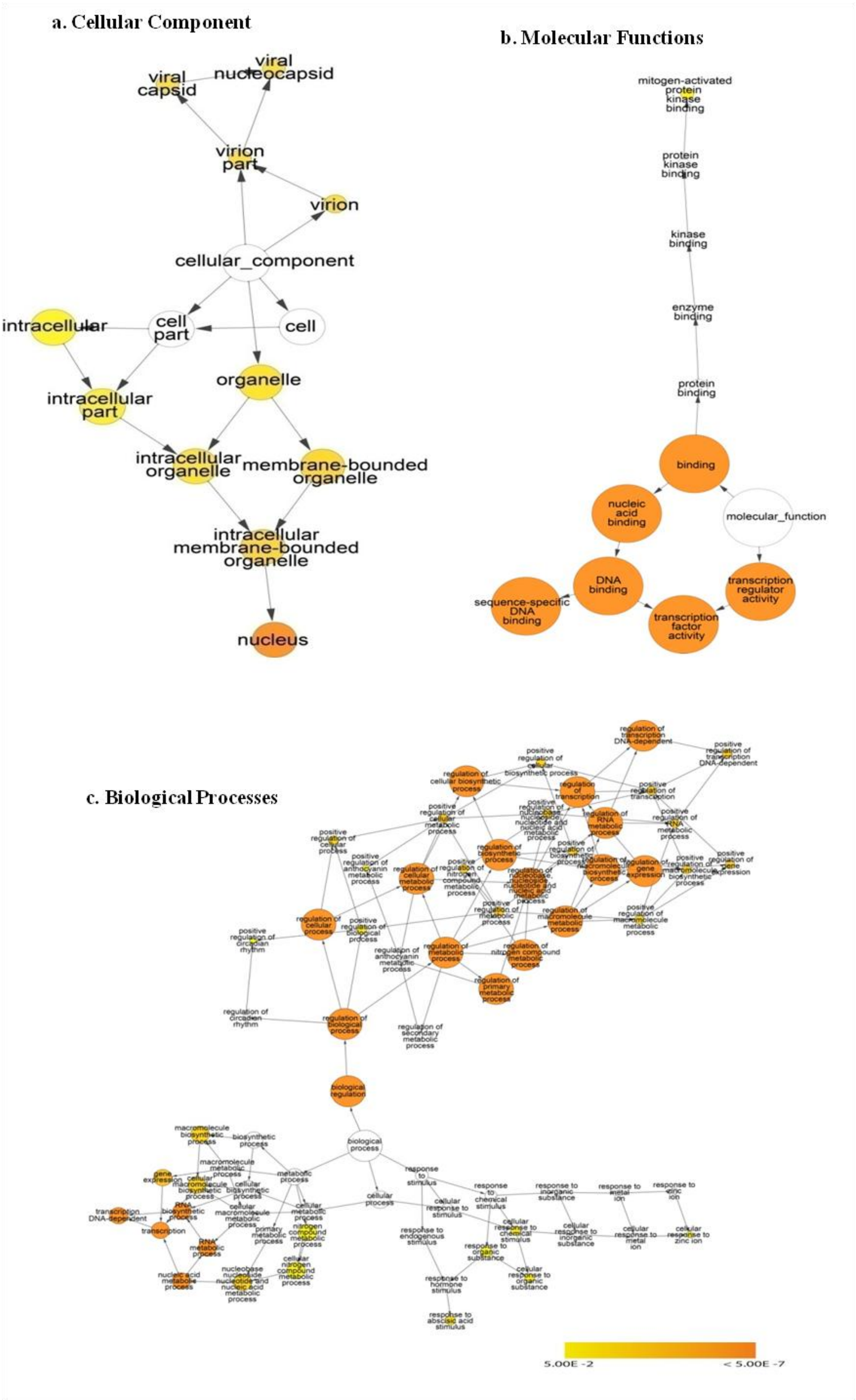

Supplementary Figure S5. Network of significantly enriched gene ontology terms for wheat bZIPs. Biological Processes (a), Cellular Components (b) and Molecular Functions (c).

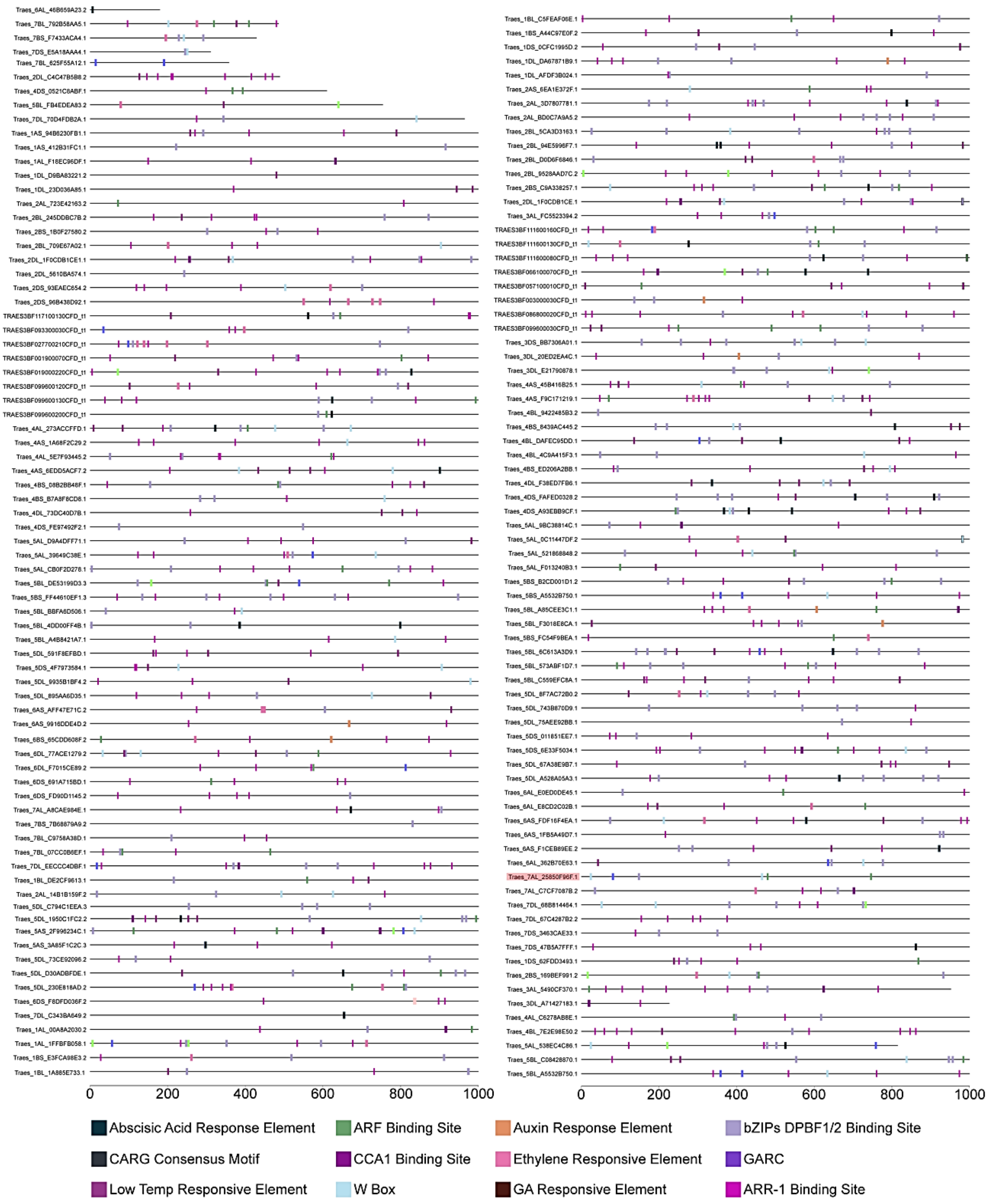

Supplementary Figure S6. 1K upstream regulatory region of wheat bZIPs and distribution of identified *cis*-elements: 12 *cis*-element distributions is shown using solid boxes of different colour background. Smaller URR are plotted for those genes which have less than 1Kb on their 5' on the contig they are present.

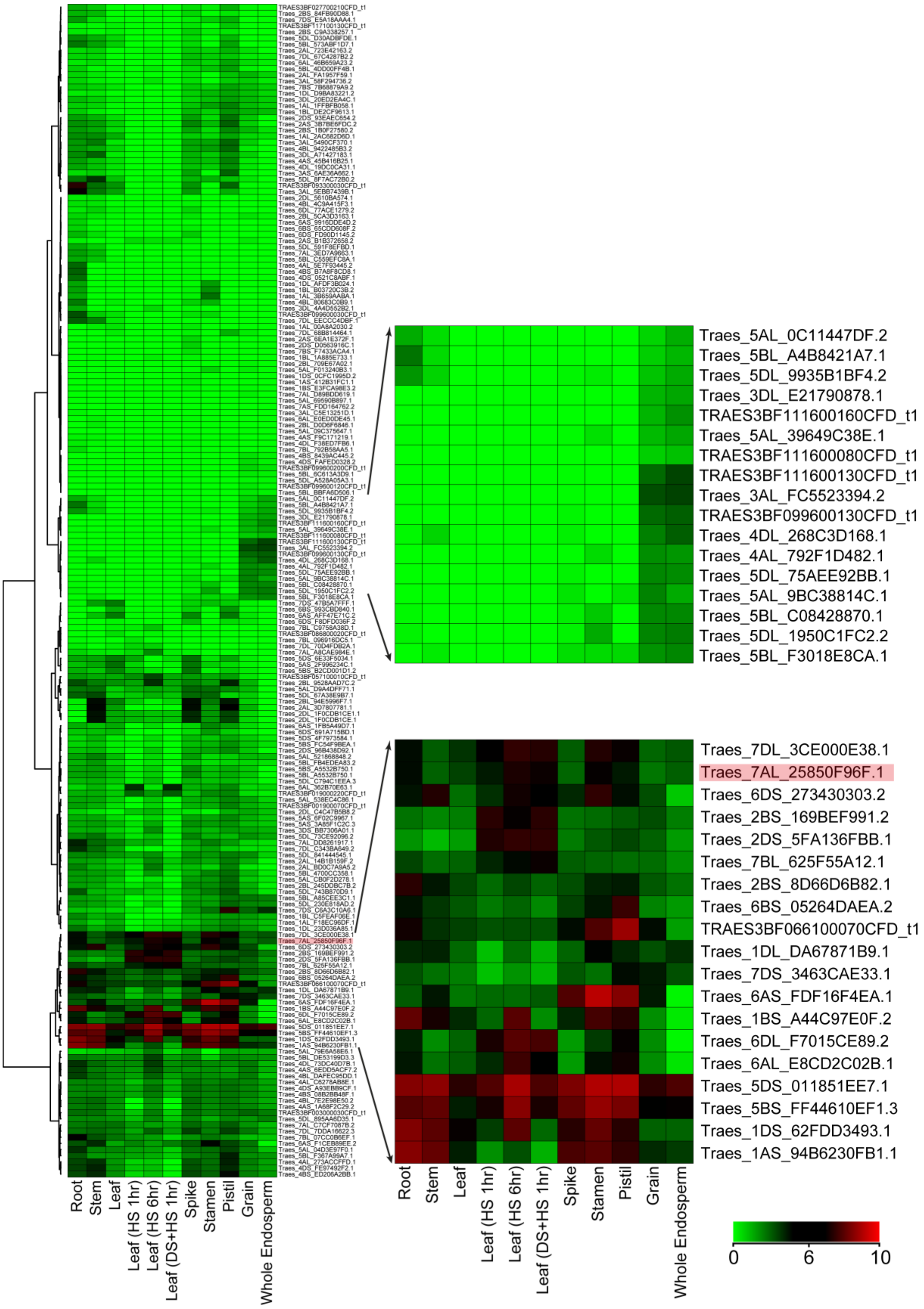

Supplementary Figure S7. Hierarchically clustered heat map showing the expression wheat bZIPs in various developmental and stressed tissues: TPM values from the ex-vip server was used to plot this heat map. Cells towards the red end display relatively higher expression while cells coloured towards the green end display relatively lower values (See text for details).

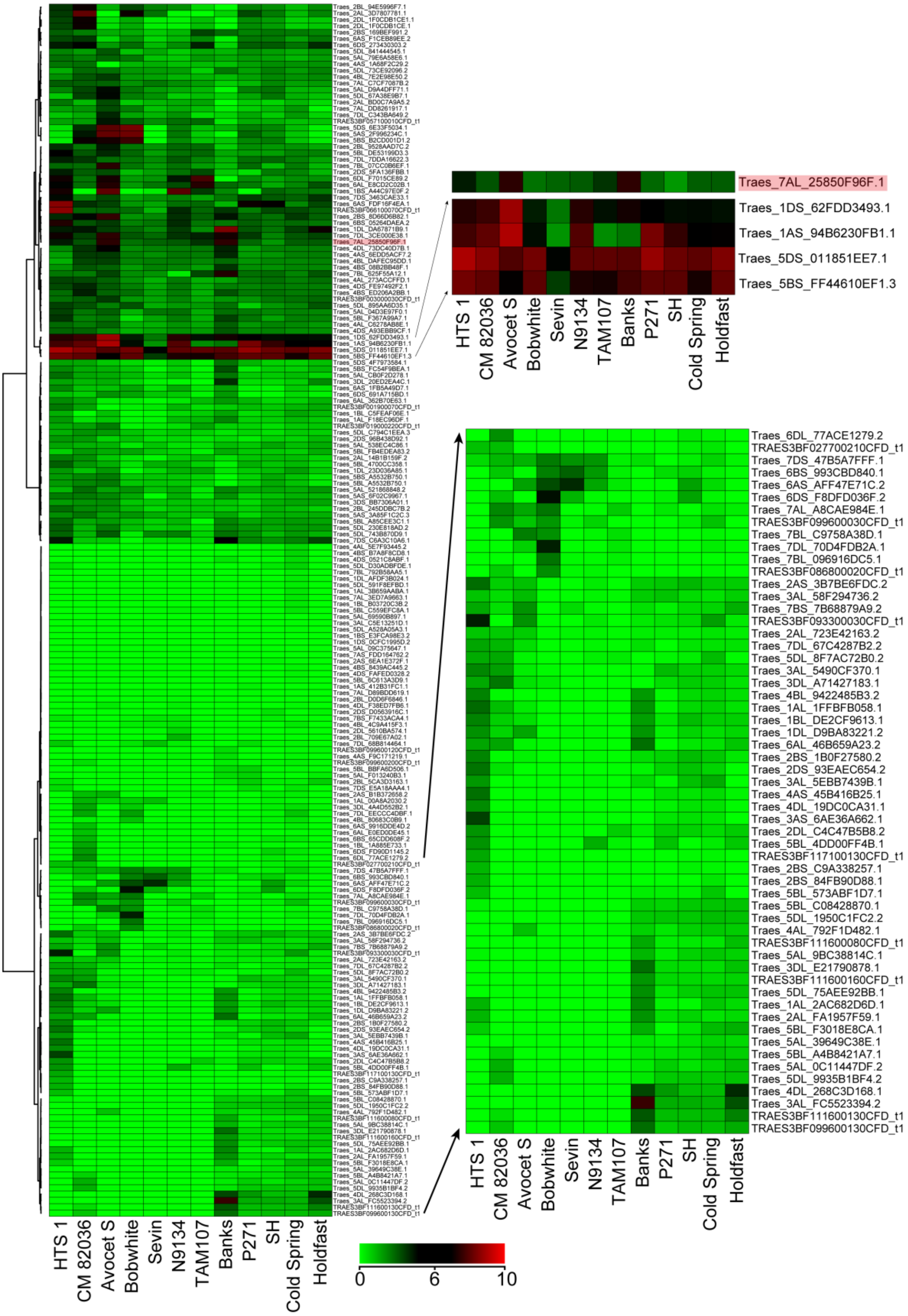

Supplementary Figure S8. Heat map showing the expression pattern of bZIPs in 12 cultivars of wheat. Heat map of hierarchically clustered and average TPM values for the all samples available in the ex vip server was used to display the relative expression level in different genotypes.

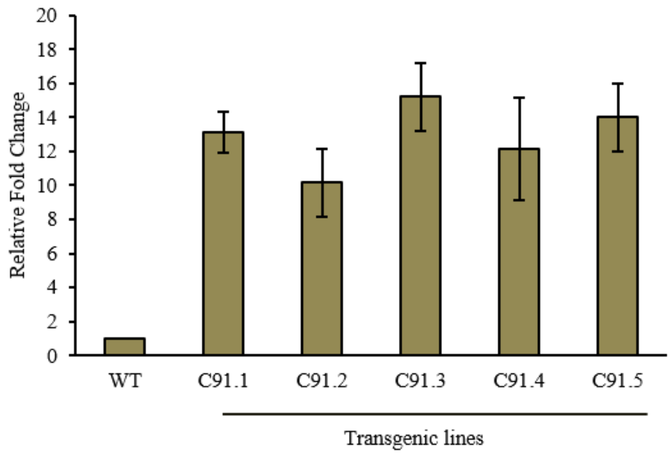

Supplementary Figure S9. The expression level of *TabZIP* in wild type (WT) and transgenic overexpressing *TabZIP*. The expression level in wild type (WT) was normalized as 1.0. The results shown are the means ± standard deviations of at least three independent experiments.

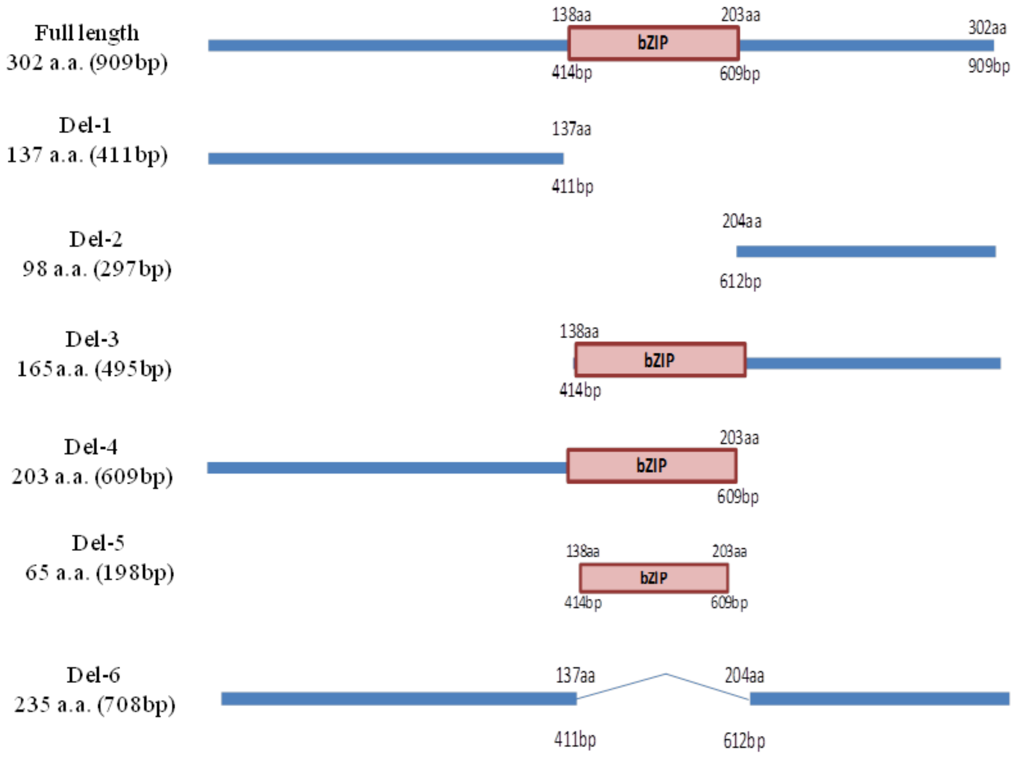

Supplementary Figure S10. Schematic representation of full length and deletions of TabZIP protein for trans-activation assay.

Supplementary Table S4. Enriched Gene Ontology cellular component category for wheat bZIPs.		
**GO-ID**	**p-value(Corrected)**	**Description**
GO-ID	corr p-value	Description
GO:0005634	1.3909E-08	nucleus
GO:0043231	0.00080606	intracellular membrane-bounded organelle
GO:0043227	0.00080606	membrane-bounded organelle
GO:0019028	0.002185	viral capsid
GO:0044423	0.002185	virion part
GO:0019012	0.002185	virion
GO:0019013	0.002185	viral nucleocapsid
GO:0043229	0.0031858	intracellular organelle
GO:0043226	0.0031858	organelle
GO:0044424	0.0093823	intracellular part
GO:0005622	0.015412	intracellular

Supplementary Table S5. Enriched Gene Ontology molecular function terms for wheat bZIPs.

**GO-ID**	**P-value (Corrected)**	**Description**
GO:0043565	0	sequence-specific DNA binding
GO:0003700	0	transcription factor activity
GO:0030528	0	transcription regulator activity
GO:0003677	0	DNA binding
GO:0003676	0	nucleic acid binding
GO:0005488	3.9232E-31	binding
GO:0051019	0.0091159	mitogen-activated protein kinase binding

Supplementary Table S6. List of primers used in the experiment.

Name	Sequence (5'-3')
AtActin_F	CTCATGCCATCCTCCGTCTT
AtActin_R	ACTTGCCCATCGGGTAATTC
AtRD29A_F	GAGCAACGAGGGGAAGATAAAAG
AtRD29A_R	TCAGTCGCACCACCACCGAACCA
AtRD29B_F	CAAAACCAAGCACCTACACA
AtRD29B_R	CTCCTTCACTCCACTTCCAC
AtERD6_F	GCAATGTGGCTGGACTGTGG
AtERD6_R	TCTTGGAGATTCGGGGATGA
AtRD20_F	ATTCGAGCACCTATGACACC
AtRD20_R	AAACTTCCATCAAAGCAACC
AtRD26_F	GGGTCGTCATCGTCTTCTTC
AtRD26_R	AACTCGGTAATCCATTGGTC
AtDREB1A-RT-F	TCCAGTTTCTTGAAACAGAGTACTC
AtDREB1A-RT-R	TCTCTAACCTCACAAACCCACTTAC
AtDREB2A-RT-F	GACCTAAATGGCGACGATGT
AtDREB2A-RT-R	TCGAGCTGAAACGGAGGTAT
AtHSP101-F	TGAGCTAGCTGTGAATGCAGGACATGCTC
AtHSP101-R	ATCACTCTTTCAGCAGATTGAGCTGCGTT
AtHsf-F	ATGGAAGAACTGAAAGTGGAAATGG
AtHsf-R	TTAAGGTTCCGAACCAAGAAAACCC
ATAF1-F	GAGTTCACGAGCGAGGTTCA
ATAF1-R	TCCACGGTGGCATCAATGTA
SOS1-F	TGCAACTCAGCATTTTCGGC
SOS1-R	AAACGAGACCTTGAGCTGGG
SOS2-F	GCATTGCCTCAGGAAGGAGT
SOS2-R	CACTTCTGCGGAAAACCACG
SOS3-F	AGGTGTCTTCCATCCAAGCG
SOS3-R	TTTCCGTCGTTTTTGCGGTC
CAT1-F	AGGAGCCAATCACAGCC
CAT1-R	TCAAGACCAAGCGACCA
CAT2-F	AACTCCGCCTGCTGTCTG
CAT2-R	ATAGGGCATCAATCCATC
CAT3-F	TCACAGCCACGCCACTAA
CAT3-R	AGAACCAAGCGACCAACC
APX1-F'	GTCCATTCGGAACAATGAGGTTTGAC
APX1-R	GTGGGCACCAGATAAAGCGACAAT
APX2-F	TGATGTGAAGACGAAGACAGGAGGAC
APX2-R	CCCATCCGACCAAACACATCTCTTA
APX3-F	CCCAAAATCACATACGCAGACCTGTA
APX3-R	AGTTGTCAAACTTCAGCGGCTCTTG
APX4-F	CTACTAAATCCGGGGGAGCCAATG
APX4-R	CTCTGTTGCATCACTCCTTCCAAAAT
APX5-F	AGCTAAACCGTCCACACAACAAAGGT
APX5-R	GTCCCAAAGTGTGACCTCCAGAGAGA
APX6 -F	TGCAAAACGAAATAAGGAAAGTGGTG
APX6-R	CACTCAGGGTTTCTGGAGGTAGCTTG
TabZIP-RT-F	ATGTTGAAGGATAGGCCTATTGGT
TabZIP-RT-R	GGTCTCTGGTTGATCACTGCTTGG

TaActin_F	GATACACGCTTCCTCATGCTATCC
TaActin_R	AGAGCCACCGATCCAGACACTG
TabZIP_F	ATGGACACCGACCTCGACCT
TabZIP_R	CTAGCAAGCGGCAGCTGCACG
TabZIP-Topo-F	CACCATGGACACCGACCTCGACCT
TabZIP-Topo-R	CTAGCAAGCGGCAGCTGCACG
TabZIP-pGBKT7- EcoR1-F	ATAGAATTCATGGACACCGACCTC
TabZIP-pGBKT7 BamH1-R	ATAGGATCCCTAGCAAGCGGCAG
TabZIP Del1_pGBKT7 EcoR1-F	ATAGGATCCGCCATCGACCTCCGTTTC
TabZIP Del1_-pGBKT7 BamH1-R	ATAGAATTCGATAGGCCTATTGGTGCTCAC
TabZIP Del2_pGBKT7 EcoR1-F	ATAGAATTCAGGTCGATGGCGATGATC
TabZIP Del3_pGBKT7 BamH1-R	ATAGGATCCAATAGGCCTATCCTTCAA
TabZIP Forward truncated _pGBKT7 EcoRI-F	ATAGAATTCCGGAGGTCGATGGCGATAGGCCTATTGGTG
TabZIP-Reverse truncated-pGBKT7 BamHI-R	ATAGGATCCCACCAATAGGCCTATCGCCATCGACCTCCG

Supplementary Data. Sequence information of *TabZIP* (Traes_7AL_25850F96F.1)

>*TabZIP*:cdna
ACGAAGCAAAAAACCCGAAGCACACGAGTCCGCTATATAACAACCGGCGGCCAGAGACCG
AGACAAAATAGCATCTCACCCCACCCATGGACACCGACCTCGACCTGGACGCCCTCCTCG
CCTCCTTCGCCGGCGAGTCCGCCGCAGTCTCCGAGCTCCTCGCCCCGCCTCCGCTCGATG
CGGCGGAGGCGGGGTCGCCGGAGTCGGTGACCTCCCGGTCCAGCCACGCCGGCGAGGAGG
TGCTGTCGGAGATCGAGAGGTTTCTGATGCAGGAGGACGAGGCGGCGGGGGCGGAGCCGG
TGGACGGGATCAGCGTGGATGAGTTCTTCGACGCGCTGTTCGATGGTGGGGAGGAGGGGG
GCGAGAAGGGGAACGGGAGTGAGGCTGAGGCTGGGGGCAGCACCGATGGGGACTCTAGGA
GGGAGGAAGAAGGGGTGGAGGTGGTGACGCCGGAGACAGAGGCGGAGGTGGTGACGCCCG
AAACGGAGGTCGATGGCGATGATCCCATCAGCAAGAAGAAGAGGAGGCAAATGAGGAATA
GGGATTCTGCCATGAAATCAAGGGAGAGGAAGAAGTCATATGTGAAGGACTTGGAGACGA
AGAGCAAGTATCTTGAGGCGGAGTGTCGCCGCCTCAGCTACGCACTTCAGTGCTGCGCAG
CTGAGAACATGGCACTGCGCCAGAACATGTTGAAGGATAGGCCTATTGGTGCTCACACAG
CCATGCAGGAGTCTGCCGTACTTTCGGAAACCCTGCCGCTGGTTTCCCTGCTTTGGCTAG
TGAGCATCGTGTGCCTATTCCTAACGCCCGGTCTACCCAACCGAAGCCTGGCGGCTCCAA
GGAGAGCCGAAAGAGGTCTCGCAATGGTAGCCGGAAAGCCAAGCAGTGATCAACCAGAGA
CCTTGGAGCTTCTACCCCATGGAAGGCGCTGGAGGGGCACAAGGGAGAGGATCAAGCTAG
ATGCTCCGCCATTGCGTGCAGCTGCCGCTTGCTAGACTAGTGCCAGCTTATTCGCAAGTT
TCCAGTATGTAGTGTAGTTAGCAATGAATGTCTAAATATGTGTTCTCTTGCTGCCACCGT
GGCTTTATCCATGACTATCTTCTGCTCTCTCTGTTCGCGTTGTTTTATGATCTTCTAATC
AGATGCTAGTTTTGAAATCTGGCGTTCTGTGTTACTTTATGTCGGGCACTCTGTGTCCAT
CTCTCTGGGTTTATGTCAAATTCAGACCATAGAAAGTTCACGAG
>*TabZIP*:cds
ATGGACACCGACCTCGACCTGGACGCCCTCCTCGCCTCCTTCGCCGGCGAGTCCGCCGCA
GTCTCCGAGCTCCTCGCCCCGCCTCCGCTCGATGCGGCGGAGGCGGGGTCGCCGGAGTCG
GTGACCTCCCGGTCCAGCCACGCCGGCGAGGAGGTGCTGTCGGAGATCGAGAGGTTTCTG
ATGCAGGAGGACGAGGCGGCGGGGGCGGAGCCGGTGGACGGGATCAGCGTGGATGAGTTC
TTCGACGCGCTGTTCGATGGTGGGGAGGAGGGGGGCGAGAAGGGGAACGGGAGTGAGGCT
GAGGCTGGGGGCAGCACCGATGGGGACTCTAGGAGGGAGGAAGAAGGGGTGGAGGTGGTG
ACGCCGGAGACAGAGGCGGAGGTGGTGACGCCCGAAACGGAGGTCGATGGCGATGATCCC
ATCAGCAAGAAGAAGAGGAGGCAAATGAGGAATAGGGATTCTGCCATGAAATCAAGGGAG
AGGAAGAAGTCATATGTGAAGGACTTGGAGACGAAGAGCAAGTATCTTGAGGCGGAGTGT
CGCCGCCTCAGCTACGCACTTCAGTGCTGCGCAGCTGAGAACATGGCACTGCGCCAGAAC
ATGTTGAAGGATAGGCCTATTGGTGCTCACACAGCCATGCAGGAGTCTGCCGTACTTTCG
GAAACCCTGCCGCTGGTTTCCCTGCTTTGGCTAGTGAGCATCGTGTGCCTATTCCTAACG
CCCGGTCTACCCAACCGAAGCCTGGCGGCTCCAAGGAGAGCCGAAAGAGGTCTCGCAATG
GTAGCCGGAAAGCCAAGCAGTGATCAACCAGAGACCTTGGAGCTTCTACCCCATGGAAGG
CGCTGGAGGGGCACAAGGGAGAGGATCAAGCTAGATGCTCCGCCATTGCGTGCAGCTGCC
GCTTGCTAG
>*TabZIP*:protein
MDTDLDLDALLASFAGESAAVSELLAPPPLDAAEAGSPESVTSRSSHAGEEVLSEIERFL
MQEDEAAGAEPVDGISVDEFFDALFDGGEEGGEKGNGSEAEAGGSTDGDSRREEEGVEVV
TPETEAEVVTPETEVDGDDPISKKKRRQMRNRDSAMKSRERKKSYVKDLETKSKYLEAEC
RRLSYALQCCAAENMALRQNMLKDRPIGAHTAMQESAVLSETLPLVSLLWLVSIVCLFLT
PGLPNRSLAAPRRAERGLAMVAGKPSSDQPETLELLPHGRRWRGTRERIKLDAPPLRAAA
AC
>*TabZIP*:Gene
ACGAAGCAAAAAACCCGAAGCACACGAGTCCGCTATATAACAACCGGCGGCCAGAGACCG
AGACAAAATAGCATCTCACCCCACCCATGGACACCGACCTCGACCTGGACGCCCTCCTCG
CCTCCTTCGCCGGCGAGTCCGCCGCAGTCTCCGAGCTCCTCGCCCCGCCTCCGCTCGATG
CGGCGGAGGCGGGGTCGCCGGAGTCGGTGACCTCCCGGTCCAGCCACGCCGGCGAGGAGG
TGCTGTCGGAGATCGAGAGGTTTCTGATGCAGGAGGACGAGGCGGCGGGGGCGGAGCCGG
TGGACGGGATCAGCGTGGATGAGTTCTTCGACGCGCTGTTCGATGGTGGGGAGGAGGGGG

GCGAGAAGGGGAACGGGAGTGAGGCTGAGGCTGGGGGCAGCACCGATGGGGACTCTAGGA
GGGAGGAAGAAGGGGTGGAGGTGGTGACGCCGGAGACAGAGGCGGAGGTGGTGACGCCCG
AAACGGAGGTCGATGGCGATGATCCCATCAGCAAGAAGAAGAGGAGGTATGCAATTTTGT
TATTTGTCGCTGGATATATATCGTCTGTCATCTAAAATTGGGCATTTTCGTTTATCTTGT
TTGCCCGTAGAACTTAGAATTTACATGAAGCTTAAGGATCAATTATCTGGAGGGTCGTTG
TCATAGTCATACTTAACCTAATCCGTAGTACTACTAAAATGCTCAAAATTGTAACAAACT
CTTAAGCTTTCAGAGATGTTGTTCCGTATGGTGAATGTTGCTCATTGGAAGAAATTTTAA
GTACATGAGGTGAGGTCACTAAAATTTTATAATTGCATAACTTATCGGTTGACATGTGCA
TAACCTTGGCTAAAAGAGCCACAATTTGTACTCCCTCCGTCCGGAAATATTTGTCATCAA
AATGAATAAAATGAGATGTATCTAGACGTATTTTAGTTCTAGATATATCTCTTTTTATCC
ATTTTGACGACAAGTATTTTCGGACGGAGGGAGTACATCTCTTGGTTAAACGGAGTCTTA
TTTTTTCTTGCTGAAAGAATCGTCTTAAATTCAAAAAAATTCTTAACCAGATGATAACTA
TCCAGTATTATTGTTTGAGAAGTAGGGATGTTTGTTTTCTCAATTGCAAGGCTATTTGGG
ACGTATAGCCTCTTACAAGTTGTGTGTTTGGTGAGTTTGTCAGGCAAATGAGGAATAGGG
ATTCTGCCATGAAATCAAGGGAGAGGAAGAAGTCATATGTGAAGGACTTGGAGACGAAGA
GCAAGTATCTTGAGGCGGAGTGTCGCCGCCTCAGCTACGCACTTCAGTGCTGCGCAGCTG
AGAACATGGCACTGCGCCAGAACATGTTGAAGGATAGGCCTATTGGTGCTCACACAGCCA
TGCAGGAGTCTGCCGTACTTTCGGGTAAGATGCTCATGGTTTTCACCATAAGCTGTATGA
ATAAAATTCTATTGCTGATGGTTTTCATTGCTCAACCATGCTGACAAACAACTAGTGCAT
TACTGTCTGGACTGCAAGTATCTCTAACGTTGGCTTGTGCTCATCTAACTTGAAATTGTT
GATATATCCATTTAAGCTTATTGGGTTATGCAACCCTCTTGCACTCAGAAAATCTAGTTT
CCTTTGATAATATCTGATCGTTTACATTAATTGAAAATAATCAGAACTTAGCGAGCTTGA
TGTCTCAGAACTTAGAGTTTGCATGAAATGCAACCTGGATTACTTGCTCGCAGAAACTTC
AGCTGTGCTAAATGTAATTGTTTTCATTTTGCAGAAACCCTGCCGCTGGTTTCCCTGCTT
TGGCTAGTGAGCATCGTGTGCCTATTCCTAACGCCCGGTCTACCCAACCGAAGCCTGGCG
GCTCCAAGGAGAGCCGAAAGAGGTCTCGCAATGGTAGCCGGAAAGCCAAGCAGTGATCAA
CCAGAGACCTTGGAGCTTCTACCCCATGGAAGGCGCTGGAGGGGCACAAGGGAGAGGATC
AAGCTAGATGCTCCGCCATTGCGTGCAGCTGCCGCTTGCTAGACTAGTGCCAGCTTATTC
GCAAGTTTCCAGTATGTAGTGTAGTTAGCAATGAATGTCTAAATATGTGTTCTCTTGCTG
CCACCGTGGCTTTATCCATGACTATCTTCTGCTCTCTCTGTTCGCGTTGTTTTATGATCT
TCTAATCAGATGCTAGTTTTGAAATCTGGCGTTCTGTGTTACTTTATGTCGGGCACTCTG
TGTCCATCTCTCTGGGTTTATGTCAAATTCAGACCATAGAAAGTTCACGAG
